# Coordination of SLC39A1 and DRP1 facilitates HCC recurrence by impairing mitochondrial quality control

**DOI:** 10.1002/ctm2.70362

**Published:** 2025-06-03

**Authors:** Rui Li, Zhe Wang, Lixin Cheng, Zhiqiang Cheng, Qiong Wu, Fengjuan Chen, Dong Ji, Qingxian Cai, Yijin Wang

**Affiliations:** ^1^ Department of Pharmacology, Joint Laboratory of Guangdong‐Hong Kong Universities for Vascular Homeostasis and Diseases, School of Medicine Southern University of Science and Technology Shenzhen China; ^2^ SUSTech Homeostatic Medicine Institute, School of Medicine Southern University of Science and Technology Shenzhen China; ^3^ Department of Critical Care Medicine, Shenzhen People's Hospital, the First Affiliated Hospital of Southern University of Science and Technology the Second Clinical Medical College of Jinan University Shenzhen China; ^4^ Department of Pathology The Second Affiliated Hospital of Southern University of Science and Technology Shenzhen China; ^5^ School of Basic Medicine North Sichuan Medical College Nanchong China; ^6^ Department of Hepatopathy The Eighth Affiliated Hospital of the Guangzhou Medical University Guangzhou China; ^7^ Senior Department of Hepatology Fifth Medical Center of Chinese PLA General Hospital Beijing China; ^8^ Department of Hepatopathy, the Third People's Hospital of Shenzhen the Second Affiliated Hospital of Southern University of Science and Technology Shenzhen China

**Keywords:** cancer therapy, HCC relapse, hepatocellular carcinoma, mitochondrial quality control

## Abstract

**Background:**

Despite rapid advances in HCC therapy, surgical resection is still the most effective treatment. However, postoperative relapse develops in a large population and the mechanism remains to be explored.

**Methods:**

HCC resection samples were retrospectively collected from 12 nonrelapsed and 15 relapsed HCC patients for RNA sequencing. Liver‐specific solute carrier family 39 member 1 (SLC39A1) knockout mice were generated by crossing Alb‐Cre mice and SLC39A1^flox/flox^ mice. Liver samples were examined for inflammation, fibrosis, proliferation, and apoptosis. Mitochondrial mass, autophagy, ROS, and mitochondrial membrane potential (MMP), were detected. Co‐immunoprecipitation and molecular docking were used to identify protein interactions.

**Results:**

SLC39A1 is highly expressed in relapsed HCC patients and negatively correlated with overall survival. Knockdown of SLC39A1 inhibited cell proliferation by arresting the cell cycle and promoted cell apoptosis, accompanied by suppressing autophagic flux. Mechanistically, SLC39A1 interacts with a member of the dynamin superfamily of GTPases dynamin‐related protein 1 (DRP1), followed by facilitating mitochondrial fission and MMP reduction. Inhibition of DRP1 abolished SLC39A1‐induced mitochondrial division and MMP depolarization, while overexpression of DRP1 reversed mitochondrial fusion and MMP hyperpolarization in SLC39A1 silenced cells, accompanied by recuperative cell proliferative ability. SLC39A1^flox/flox^,Alb‐Cre mice displayed fewer tumour numbers and less liver damage compared with SLC39A1^flox/flox^ mice. A specific peptide targeting SLC39A1 to disturb the combination of full‐length SLC39A1 and DRP1 efficiently suppressed HCC progression.

**Conclusions:**

Our findings reveal a key role of SLC39A1‐DRP1 interaction in HCC progression by disturbing mitochondrial quality control and providing a competitive peptide as a potential anti‐tumour therapy.

**Key points:**

- SLC39A1 correlates with HCC recurrence and HCC mortality.
- Interaction of SLC39A1 and DRP1 facilitates HCC by regulating mitochondrial quality control.
- Specific peptide targeting SLC39A1 efficiently prevents HCC progression.

## 1 INTRODUCTION

Hepatocellular carcinoma (HCC) is one of the most common malignant tumours, ranking as the second leading contributor to global cancer mortality rates.^1^ Despite the rapid development of targeted therapy, surgical resection remains the most effective treatment for HCC, especially at early stages.^2^ Surgical resection provides better clinical outcomes than other potentially curative therapies, particularly among patients with well‐preserved hepatic function.^3^ However, postoperative HCC relapse developed in approximately 70% of patients at 5 years and is associated with low survival.^4^ Identification of patients after surgery with a high risk of recurrence allows patients to provide better surveillance timely. More importantly, the biology of hepatocarcinogenesis derived from residual cancer cells remained to be elucidated. Adjuvant therapy targeting this biological process in surgically treated patients may reduce the risk of relapse and improve survival.^5^

Autophagy and metabolic reprogramming are currently considered vulnerabilities in multiple malignancies.^6^ Autophagy favours cancer cells to survive under hypoxic stress by preventing the production of reactive oxygen species (ROS) and decreasing apoptosis, critically contributing to tumour maintenance and progression.^7^ Moreover, autophagy activation was observed in HCC patients, indicating that therapeutic interventions targeting these activated signalling pathways with promising clinical implications.^8^ However, evidence demonstrate autophagy suppresses tumorigenesis by mitigating metabolic stress, in concert with apoptosis.^9^ Clinically aggressive malignant HCC cell lines and tumour specimens from patients with disease recurrence demonstrate significantly reduced autophagic activity compared with these less malignant cell lines or primary tissues.^10^ Evidence indicates enhanced autophagy may exert tumour‐suppressive effects during premalignant disease stages.^11^

Mitochondria are highly dynamic organelles in maintaining cell homeostasis, whereas defects in mitochondrial dynamics underlie many pathologies.^12^ The dynamin‐related protein 1 (DRP1), encoded by DNM1L, plays a central role in controlling mitochondrial fission, a critical step required for initiating mitophagy.^13^ Alteration of DRP1 caused by reduction of mitochondrial membrane potential (MMP) could induce mitophagy, for eliminating dysfunctional mitochondria.^14^ These molecular modifications facilitate the recruitment of autophagic adaptor proteins, mediating autophagosome biogenesis and culminating in selective mitochondrial degradation.^15^ Additionally, considering that DRP1‐dependent mitochondrial fission is essential for mitophagy initiation, suppressing mitophagy by targeting DRP1 possibly increases tumour cell apoptosis in the adaption to hypoxia, indicating a novel therapeutic approach through mitochondrial homeostasis modulation.^16^

Herein, we identified a molecule SLC39A1 that is a molecule overexpressed in relapsed HCC patients and has inverse correlations with overall survival. Our findings indicate that SLC39A1 promoted cellular proliferation and migration while repressing cell apoptosis, accompanied by activated autophagy. Furthermore, the interaction of SLC39A1 and DRP1, accompanied by MMP reduction, facilitates mitochondrial fission and perturbs mitochondrial quality surveillance, which contributes to HCC progression. A specific peptide targeting SLC39A1 to disturb its interaction with DRP1 efficiently suppressed HCC progression in vivo and in vitro. Our findings provide mechanistic insights into hepatocarcinogenesis and provide a competitive peptide as a potential anti‐tumour therapy.

## 2 MATERIALS AND METHODS

### 2.1 Patient tissue

Liver specimens from 12 nonrelapsed and 15 relapsed HCC patients undergoing curative surgical resection at Fifth Medical Center of Chinese PLA General Hospital from June 2012 to April 2016 (Table S1, cohort 1) were continuously collected for RNA sequencing. Matched tumour and adjacent tissue pairs from 5 non‐relapsed and 6 relapsed HCC patients who underwent surgical resection at Shenzhen Third People's Hospital from February 2021 to May 2022 (Table S2, cohort 2) were used for western blotting analysis of protein expression. All patients were pathologically diagnosed with HCC and followed up for prognostic assessment. No patient had received immunotherapy before surgery. This research received formal authorization from the local Ethical Committee (license number: 2020055D and 2024–181).

### 2.2 Animal models

Mice were maintained under SPF conditions. All experimental procedures involving animal subjects were conducted in compliance with international animal welfare guidelines (license number: JY202304004) and authorized approved by the Institutional Animal Care and Use Committee of Southern University of Science and Technology. The SLC39A1 gene, mapped to chromosome 3 (GRCm39), comprises four exons with exons 2–3 strategically selected for Cre‐loxP‐mediated conditional deletion to achieve functional gene ablation. Liver‐specific SLC39A1 knockout mice (SLC39A1^flox/flox^,Alb‐Cre) were generated via successive crosses between SLC39A1^flox/flox^ heterozygotes and commercially sourced Alb‐Cre transgenic lines (Cyagen Biosciences). Both sexes were included in experimental cohorts.

To develop a fibrosis‐associated HCC model, a staged chemical induction protocol was implemented. Mice received intraperitoneal administration of diethylnitrosamine (DEN, 50 mg/kg, MedChemExpress) during the early postnatal phase (14–16 days). Subsequently, carbon tetrachloride (CCl4, 5 mL/kg) was potentiated through biweekly intraperitoneal injections until 19 weeks of age to promote HCC progression.

For subcutaneous tumour model, 2 × 10^6^ logarithmic growth‐phase Hepa1‐6 cells in 100 µL 1:1 mixture of serum‐free high glucose DMEM (Macgene) and Matrigel (Yeasen) were injected into axillary regions of the BALB/C nude mice (GemPharmatech). Tumour dimensions were quantified through calliper measurements, with volume derived using the standard formula (length × width^2^)/2.

### 2.3 Cell lines

Human cell lines Huh7 (Type Culture Collection of the Chinese Academy of Sciences, cat. SCSP‐526), HepG2 (Servicebio, cat. STCC10114P), HCCLM3 cells (Servicebio, cat. STCC10111P), MHCC97H (Servicebio, cat. STCC10113P) and mice cell line Hepa1‐6 cells (Servicebio, cat. STCC20016P) were maintained in DMEM (Macgene) containing 10% FBS (VivaCell) and 1% penicillin‐streptomycin (Macgene), with all media components filter‐sterilized before use. All cells were grown in DMEM under a temperature‐controlled incubator at 37°C with a 5% CO_2_ environment. More information is provided in Supplementary materials.

## 3 RESULTS

### 3.1 SLC39A1 expression in resected tumours is higher in recurrent patients than in non‐recurrent patients

**FIGURE 1:**
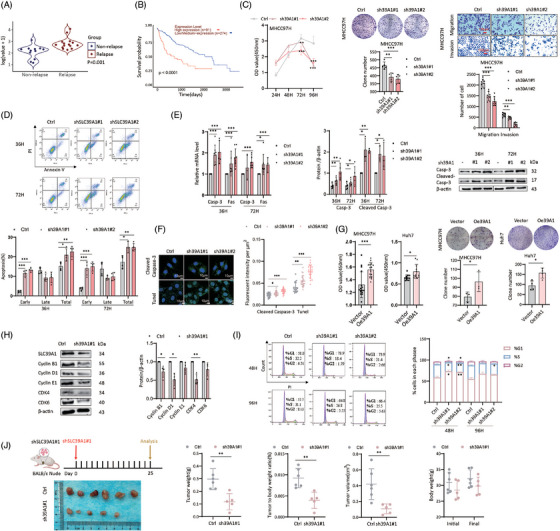
SLC39A1 is highly expressed in HCC‐relapsed patients, promotes cell proliferation and migration and impedes apoptosis in vitro. (A) SLC39A1 mRNA expression in 12 non‐relapsed and 15 relapsed HCC patients in our HCC cohort. (B) Kaplan–Meier curves of overall survival of HCC patients according to the level of SLC39A1 in the TCGA dataset. (C) Cell proliferation, cell clonal formation, and cell migration and invasion analysis in control and SLC39A1‐knockdown cells. (D) Cell apoptosis analysis was determined by flow cytometry with PI/Annexin V staining. (E) mRNA expressions of Casp‐3 and FAS, protein levels of Casp‐3 and Cleaved Casp‐3 in control and SLC39A1‐knockdown cells. (F) Immunofluorescent staining for Cleaved Casp‐3 and Tunel in control and SLC39A1‐knockdown cells. (G) Analysis of cell proliferation and clonal formation in control and SLC39A1 overexpressed cells. (H) Protein levels of Cyclin B1, D1, E1 and CDK4, CDK6 were detected in control and SLC39A1‐knockdown cells. (I) The percentage of the G1 phase, S phase and G2 phases of the cell cycle were detected by flow cytometry in control and SLC39A1‐knockdown cells. (J) Control and SLC39A1‐knockdown cells were implanted subcutaneously in nude mice. The results of the statistical analysis of tumour weight, tumour volume and body weight are shown on the right (control, *n *= 6; shSLC39A1, *n* = 5). Data are expressed as mean ± SD (*n* ≥ 3). ***p < .001, **p < .01, and *p < .05.

A total of 78 significant differentially expressed genes (DEGs) were identified in 15 HCC relapsed groups compared with 12 non‐relapsed groups after resection (Figure S1A). Although 22 of 27 HCC patients were male, no sex‐specific disparity in recurrence rates was detected between male and female patients (Table S1). RNA sequencing transcriptome analysis by KEGG and GO revealed that HCC recurrence was related to neutrophil degranulation and apoptosis (Figure S1B). Hallmark pathways contained xenobiotic metabolism and oxidative phosphorylation in the relapsed group (Figure S1C). There was a moderate increase in the infiltrating immune cells in non‐relapsed compared with relapsed patients, although no significant difference was observed (Figure S1D–F). Notably, we found SLC39A1 and SLC66A3, but not SLC50A1, were significantly upregulated in HCC‐relapsed patients and negatively associated with overall survival (OS) in the TCGA database (Figure 1A,B; Figure S2A–C). The proportion of neutrophil cells was moderately increased in patients with high expression of SLC39A1, SLC50A1, and SLC66A3, respectively, than that with low expression (Figure S2D). In vitro, all three SLCs were highly expressed in MHCC97H cells, suggesting expression of all three SLCs correlates with the degree of HCC malignancy (Figure S3A). We analyzed the expressions of SLC39A1, SLC50A1 and SLC66A3 across tumour stages from Stage I to Stage IV in the TCGA database. The data showed that SLC39A1 expression levels were elevated in patients with advanced‐stage HCC compared with those with early‐stage HCC, although lacking statistical difference (Figure S3B). We also analyzed RNA sequencing data in HCC patients with or without HCC metastasis (GSE158408, GSE245587). However, no significant difference in SLC39A1 expression levels was observed between metastatic and non‐metastatic HCC patients (Figure S3C).

### 3.2 SLC39A1 promotes cell proliferation and inhibits cell apoptosis

To investigate the role of the three SLCs in HCC proliferation, short hairpin RNA (shRNA) specifically targeting SLC39A1, SLC50A1, and SLC66A3 were constructed in the MHCC97H cell line (Figure S3D). Depletion of SLC39A1, but not SLC50A1 and SLC66A3, resulted in a dramatically decreased cell proliferation rate (Figure 1C; Figure S3E). Knockdown of SLC39A1 achieved significant reductions in both clonogenicity, cell migration and cell invasion, while SLC66A3 only corresponded to reduced clonogenicity and SLC50A1 only corresponded to reduced cell migration (Figure 1C; Figure S3F,G). Apoptosis plays a critical role in HCC, influencing tumour progression, therapeutic resistance, and survival pathways.^17^, ^18^, ^19^ Knockdown of SLC39A1 also stimulated apoptosis, evidenced by upregulation of Fas cell surface death receptor (Fas), Caspase‐3 (Casp‐3), and cleaved Casp‐3 (Figure 1D–F; Figure S3H,I). Consistently, silencing SLC39A1 effectively attenuated malignant phenotypes in HepG2 cells (Figure S4A–F). SLC39A1 overexpression promoted cell proliferation and colony formation in MHCC97H and Huh7 cell lines (Figure 1G; Figure S4G). Notably, the knockdown of SLC39A1 arrested the cell cycle to the G1 phase through the downregulation of Cyclin‐dependent kinase4 (CDK4), Cyclin B and Cyclin D, which contributed to DNA synthesis and DNA transition into the S phase (Figure 1H,I).

Nude mice bearing subcutaneous tumours were used to investigate the role of SLC39A1 on tumour growth in vivo (Figure 1J; Figure S4H). As a murine‐derived cell line, Hepa1‐6 cells exhibit higher tumorigenic capacity than human‐derived HCC cell lines in the subcutaneous xenograft nude mice model.^20^ Our prior experiments demonstrated that MHCC97H cells, being of human origin, show poor tumour formation efficiency in the subcutaneous xenograft nude mice model. To ensure robust tumour growth in vivo, we selected Hepa1‐6 cells to do further tumorigenic experiments in mice. Consistent with the in vitro result, the knockdown of SLC39A1 attenuated tumour progression, including reduced tumour weight, tumour‐to‐body weight ratio and tumour volume, while body weight was barely changed (Figure 1J). Collectively, SLC39A1 is demonstrated as a driver in HCC progression by both increasing malignant phenotypes and inhibiting cell apoptosis.

### 3.3 Cellular Zn^2+^ is not involved in SLC39A1 mediated cell proliferation and migration

**FIGURE 2:**
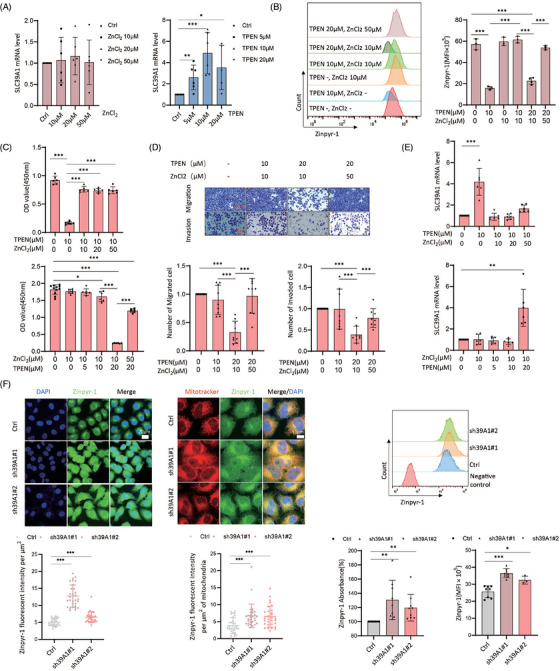
Cellular Zn^2+^ level is correlated with cell proliferation and migration in MHCC97H cells but not involved in SLC39A1‐mediated cell proliferation and migration. (A) SLC39A1 mRNA expression in MHCC97H cells treated with ZnCl_2_ or TPEN. (B) Analysis of cellular Zn^2+^ levels in MHCC97H cells treated with different combinations of ZnCl_2_ and TPEN by flow cytometry. (C, D) Cell proliferation, migration and invasion analysis in MHCC97H cells treated with different combinations of ZnCl_2_ and TPEN. (E) SLC39A1 mRNA expression in MHCC97H cells treated with different combinations of ZnCl_2_ and TPEN. (F) Intracellular and mitochondrial Zn^2+^ levels detected by Zinpyr‐1 (1 µM) in MHCC97H cells by Immunofluorescent staining, microplate reader and flow cytometry. Negative control cells represent no Zinpyr‐1 staining in flow cytometry. Data are expressed as mean ± SD (*n* ≥ 3). ***p < .001, **p < .01, and *p < .05.

SLC39A1 acts as a zinc ion (Zn^2+^) transporter responsible for the uptake of Zn^2+^ into cells to maintain Zn^2+^ homeostasis.^21^ Exogenous Zncl_2_ or Zn^2+^ chelator, N, N, N’, N’‐tetrakis (2‐pyridylmethyl) ethylenediamine (TPEN), were used to increase or reduce intracellular Zn^2+^, respectively. Cell proliferation was markedly enhanced with Zncl_2_ treatment at 20 and 50 µM, but decreased at 500 µM (Figure S5A). Conversely, TPEN treatment led to substantial cell death at concentrations of 10 and 20 µM (Figure S5B). In addition, Zn^2+^ deficiency induced by TPEN attenuated cell migration and invasion, while the aforementioned Zncl_2_ treatment does not alter these capabilities (Figure S5C,D). Metal regulatory transcription factor 1 (MTF‐1), zinc sensing receptor (ZnR/GPR39), metallothionein (MTs) and zinc‐regulated GTPase metalloprotein activator 1 (ZNG1) are key elements in maintaining a strict intracellular Zn^2+^ homeostasis. Zn^2+^ depletion led to an upregulation of SLC39A1, ZNR, MT1A, and MT1B in MHCC97H cells, but Zncl_2_ addition did not change these regulators (Figure 2A; Figure S5E), suggesting depletion of Zn^2+^ seemed to trigger a compensatory response to maintain Zn^2+^ homeostasis. Zncl_2_ completely abolished the inhibitory effect of TPEN on cellular Zn^2+^ level, cell proliferation and migration (Figure 2B–D). On the contrary, the upregulation of SLC39A1, SLC30A1, MT1B, ZNG1C, ZNG1E, and ZNG1F under Zn^2+^ deficient condition was prevented by additional Zncl_2_ (Figure 2E; Figure S6A–D).

We next studied whether intracellular zinc mediates SLC39A1‐associated cell proliferation. Transcription of MTF‐1 and MT1A was increased in SLC39A1 silenced cells (Figure S5F). However, the knockdown of SLC39A1 led to an overabundance of intracellular and mitochondrial Zn^2+^ (Figure 2F). Based on our previous finding that the Zn^2+^ deficiency attenuated cell proliferation, we hypothesized alteration of Zn^2+^ is not involved in SLC39A1 promoting HCC.

### 3.4 SLC39A1 inhibits cell apoptosis medicated by triggering autophagy

**FIGURE 3:**
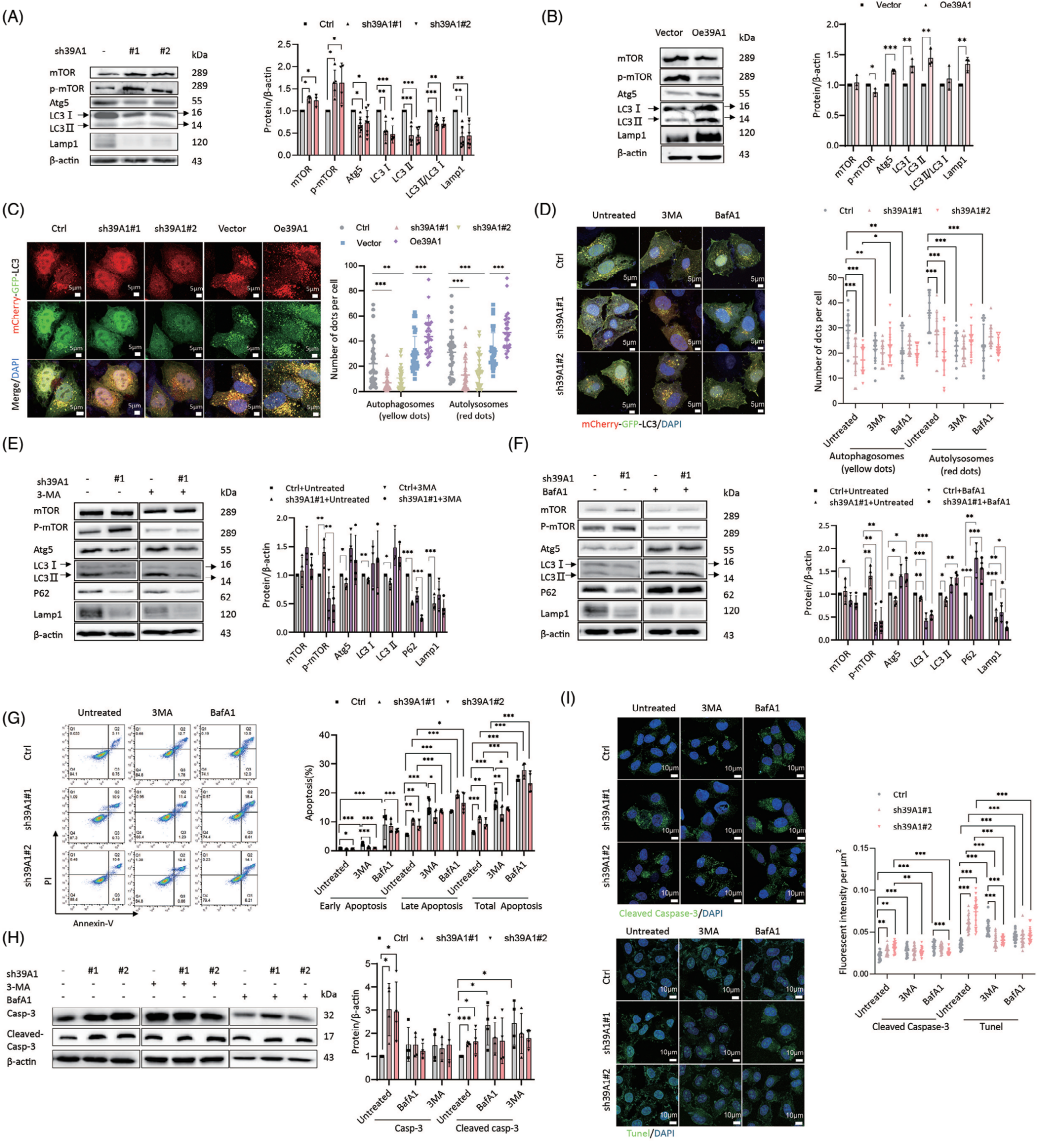
SLC39A1 inhibits cell apoptosis medicated by triggering autophagy. (A, B) Western blot analysis of mTOR, Atg5, LC3 and Lamp1 proteins in SLC39A1 silenced and overexpressed cells. (C) mCherry‐GFP‐LC3 adenovirus infection by transfection plasmid to detect autophagosomes (yellow dots) and autolysosomes (red dots) formations in SLC39A1 silenced and overexpressed cells. (D) The autophagosomes (yellow dots) and autolysosomes (red dots) formations in SLC39A1 silenced with or without 3‐methyladenine (3‐MA) (1 µM, 48H) and bafilomycin A1 (BafA1) (1 µM, 48H) treatments (*n* = 15–30 cells). (E, F) Western blot analysis of mTOR, Atg5, LC3, P62 and Lamp1 proteins in SLC39A1 silenced cells with or without 3MA (1 µM, 48H)/BafA1 (1 µM, 48H) treatment. (G) Analysis of cell apoptosis by flow cytometry with PI/Annexin V staining in SLC39A1 silenced cells with or without 3MA (1 µM, 48H)/BafA1 (1 µM, 48H) treatment. (H) Western blot analysis of Casp‐3 and Cleaved Casp‐3 expressions in SLC39A1 silenced cells with or without 3MA (1 µM, 48H)/BafA1 (1 µM, 48H) treatment. (I) Immunofluorescent staining for cleaved Casp‐3 and Tunel in SLC39A1 silenced cells with or without 3MA(1 µM, 48H)/BafA1(1 µM, 48H) treatment. Data are expressed as mean ± SD (*n* ≥ 3). ***p < .001, **p < .01, and *p < .05.

Autophagy generally contributes to tumour progression due to microenvironment remodelling under tumour stress.^22^ We studied whether SLC39A1 participates in autophagic activity. SLC39A1 deletion displayed increased mammalian target of rapamycin (mTOR), p‐mTOR and decreased autophagy related 5 (Atg5), microtubule‐associated protein 1 light chain 3 (LC3), lysosomal‐associated membrane protein 1 (Lamp1) in MHCC97H cells (Figure 3A). Meanwhile, Atg5, LC3 and Lamp1 were upregulated in SLC39A1 overexpressed cells (Figure 3B). These results indicated that SLC39A1 played an important role in autophagosome synthesis. MCherry‐GFP‐LC3 adenovirus was used to recognize autophagosome and autolysosome formations in MHCC97H cells. Knockdown of SLC39A1 significantly decreased the number of autophagosomes and autolysosomes, and SLC39A1 overexpression promoted the formation of autophagosomes and autolysosomes, confirming the role of SLC39A1 in autophagic process (Figure 3C).

We then used 3‐methyladenine (3‐MA) to suppress autophagosome formation. No obvious inhibition of autophagosome and autolysosome formations was detected in SLC39A1 silenced cells treated with 3‐MA (Figure 3D), evidenced by decreased p‐mTOR, and increased Atg5 and LC3 (Figure 3E). Similarly, treatment with bafilomycin A1 (BafA1), which blocked autophagic flux, also reversed the decrease of autophagosome and autolysosome formations (Figure 3D), accumulation of p‐mTOR and suppression of Atg5 and LC3 proteins in SLC39A1‐knockdown cells (Figure 3F). Taken together, SLC39A1 enhanced autophagosome formation at an earlier stage and degradation in acidic lysosomes, thereby promoting the autophagic process.

We next determined whether autophagy is involved in SLC39A1‐mediated cell proliferation and migration. Treatment with 3‐MA failed to reverse the inhibitory effect of cell proliferation, migration and cell apoptosis in cells with genetic ablation of SLC39A1 (Figure S7A,B). Although BafA1 significantly reduced cell proliferative and migration, without altering the suppressive impact of shSLC39A1 on cell proliferation and migration (Figure S7C,D). Both 3‐MA and BafA1 abolished the upregulated apoptosis in SLC39A1 silenced cells (Figure 3G–I), indicating that SLC39A1 inhibits cell apoptosis medicated by triggering autophagy.

### 3.5 SLC39A1 promotes mitochondrial fission accompanied by reduction of mitochondrial membrane potential

**FIGURE 4:**
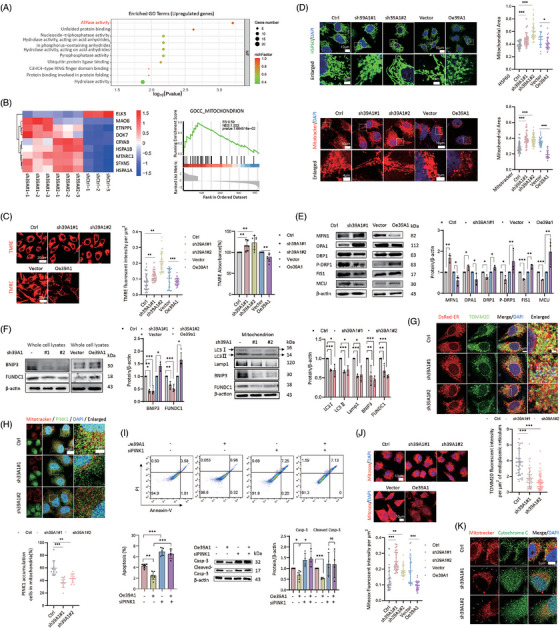
SLC39A1 induces mitochondrial fission and enhanced mitophagy accompanied by reduction of mitochondrial membrane potential. (A) RNA‐seq analysis in SLC39A1‐knockdown cells and control cells and DEGs were enriched in adenosine triphosphatase (ATPase) activity. (B) The DEGs were enriched in mitochondrion by GO analysis. (C) The MMP levels in SLC39A1 silenced and overexpressed cells using TMRE dye by Immunofluorescent staining and a multimode microplate reader (*n* = 30 cells). (D) Immunofluorescent analysis of mitochondrial morphology by HSP60 antibody and Mitotracker staining. (E) Western blot analysis of DRP1, FIS1, MFN1 and OPA1 expression in SLC39A1 knockdown and overexpressed cells. (F) Western blot analysis of BNIP3 and FUNDC1 expression in whole cell lysates and isolated mitochondria. (G) Immunofluorescent staining for TOMM20 antibody and DsRed‐ER in MHCC97H cells (n≥30 cells). (H) Immunofluorescent staining for PINK1 and Mitotracker staining in MHCC97H cells (*n* = 90–150 cells). (I) Analysis of cell apoptosis by flow cytometry with PI/Annexin V staining and western blot analysis of Casp‐3 and Cleaved Casp‐3 expressions in SLC39A1 overexpressed cells treated with genetic inhibition of PINK1 (siPINK1). (J) Intracellular ROS detected by MitoSox Red staining in MHCC97H cells (*n* = 30 cells). (K) Immunofluorescent analysis of Cytochrome C and Mitotracker staining. The white arrows indicate the released cytochrome C. Data are expressed as mean ± SD (*n* ≥ 3). ***p < .001, **p < .01, and *p < .05.

Herein, comparative RNA sequencing transcriptomics was performed to identify downstream pathways between SLC39A1‐knockdown cells and control cells (Figure S8A,B). Intersected DEGs were enriched in adenosine triphosphatase (ATPase) activity (Figure 4A), MAPK signalling pathway, cell cycle and ion transport pathway (Figure S8C–H). Mitochondrion was regarded as a key cellular component for the biological function of SLC39A1 (Figure 4B). We found that SLC39A1 was partially localized in the mitochondrion (Figure S9A). Mitochondrial membrane potential (MMP) level was elevated in SLC39A1 silenced cells and reduced in SLC39A1 overexpressed cells, suggesting MMP regulated by SLC39A1 (Figure 4C).

Given that SLC39A1 is located in the mitochondrion and leads to MMP depolarization, it seemed to be involved in mitochondrial morphology. A notable reduction of punctate mitochondria and an increase of elongated mitochondria were observed in SLC39A1‐silenced cells (Figure 4D; Figure S9B,C), consistent with a markedly upregulated OPA1 and MFN1, downregulated FIS1 and DRP1 (Figure 4E; Figure S9D,E). Consistently, increased mitochondrial fragmentation and the declined mitochondrial fusion were noted in SLC39A1 overexpressed cells, accompanied by upregulated FIS1 and DRP1 and downregulated OPA1 and MFN1 (Figure 4E). These results indicated that SLC39A1 can induce mitochondrial fission, which may partially account for the MMP reduction.

### 3.6 SLC39A1 induces mitophagy to inhibit the generation of ROS

PTEN‐induced putative kinase protein‐1 (PINK1) coordinates mitochondrial fission and Bcl2 interacting protein 3 (BNIP3)/FUN14 domain‐containing 1 (FUNDC1)‐mediated mitophagy by recruiting LC3 via ubiquitin phosphorylation or direct receptor interaction, thereby selectively eliminating damaged mitochondria and maintaining cellular homeostasis.^23^ SLC39A1 deletion displayed reduced BNIP3 and FUNDC1 (Figure 4F), consistent with inhibition of LC3 and Lamp1 in isolated mitochondria from SLC39A1‐knockdown cells, suggesting impaired mitophagy in SLC39A1 deficient cells. Previous studies demonstrated that autophagosome formation occurs at mitochondria‐ER contact sites which are known to be affected by mitochondrial fusion and fission.^24^ The autophagosome formation was suppressed at the ER–mitochondria contact site in SLC39A1‐silenced cells, indicating that SLC39A1 is involved in autophagosome formation at the ER–mitochondria contact site (Figure 4G). We evaluated the recruitment of PINK1 and Parkin at OMM. SLC39A1 deletion inhibited the accumulation of PINK1 on OMM, subsequently leading to the reduced recruitment of Parkin to damaged mitochondria (Figure 4H; Figure S9F). P62 is an autophagic adapter protein, which is recruited to depolarized mitochondria after Parkin‐directed ubiquitylation to recognize and clear dysfunctional mitochondria. SLC39A1 deletion markedly suppressed the accumulation of P62 in mitochondria (Figure S9F). Knockdown of PINK1 abolished the downregulated apoptosis in SLC39A1 overexpressed cells (Figure 4I; Figure S9G), indicating PINK1 is involved in cell apoptosis medicated by SLC39A1. We hypothesized that impaired mitophagy was linked to oxidative stress mediated by ROS production. ROS triggered cell death by facilitating the release of cytochrome C (Cyto‐C) from mitochondria, subsequently triggering the activation of casp‐3 and initiating cell apoptosis. Supporting our hypothesis, the absence of SLC39A1 resulted in increased levels of ROS and release of Cyto‐C (Figure 4J,K; Figure S9H,I).

### 3.7 SLC39A1/DRP1/MCU axis contributes to MMP reduction and mitochondrial fragmentation

**FIGURE 5:**
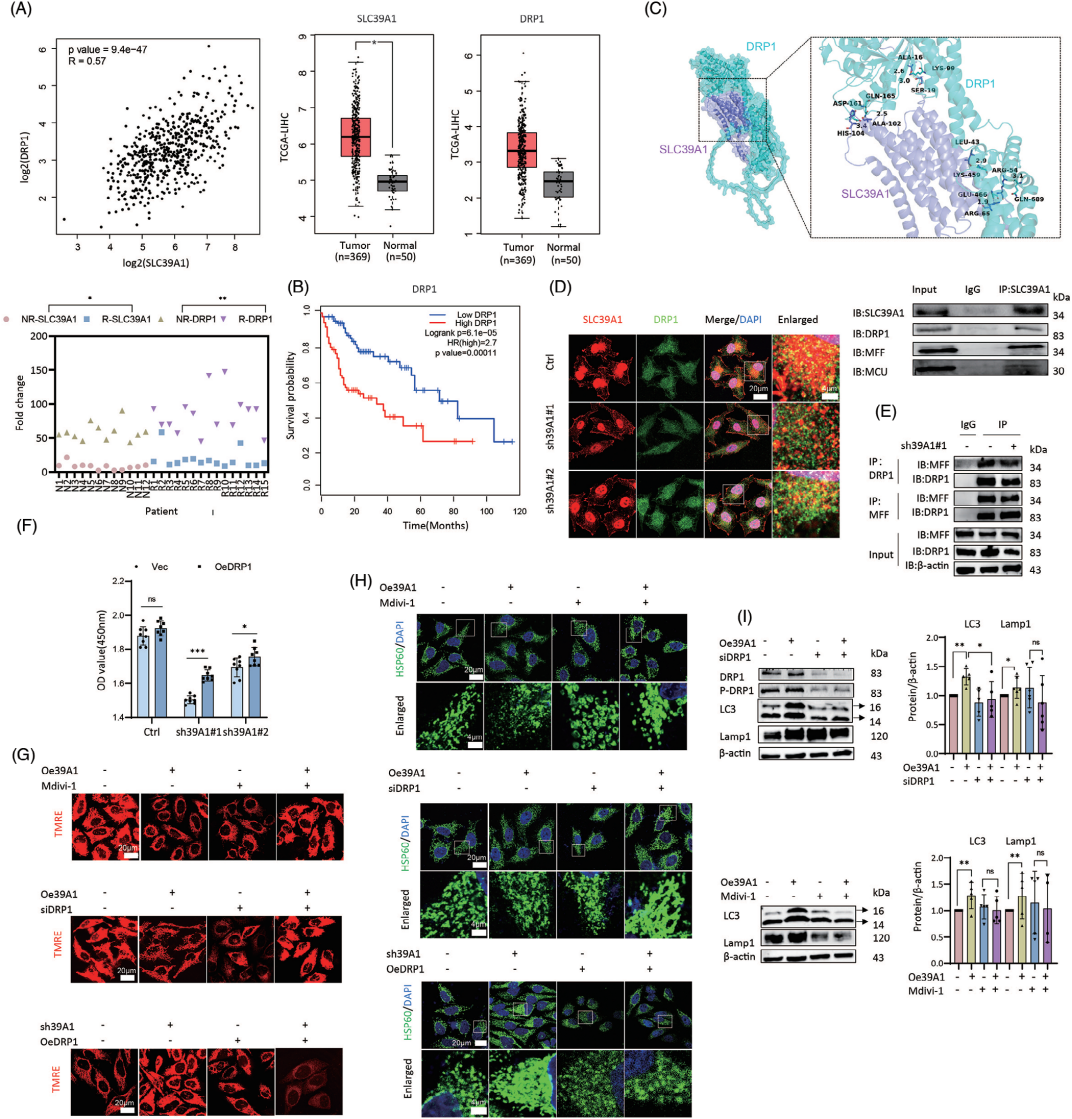
SLC39A1/DRP1/MCU axis contributes to MMP reduction and mitochondrial fragmentation. (A) The correlation between SLC39A1 and DRP1 expressions in HCC patients in TCGA‐LIHC dataset, and their expressions in tumour versus normal tissues derived from TCGA‐LIHC dataset, and in relapsed versus non‐relapsed HCC tumours from our HCC cohort. Non‐relapsed: NR; relapsed: R. (B) Kaplan–Meier survival analysis of HCC patients with high or low DRP1 expression derived from TCGA‐LIHC dataset. (C) Physical interaction between SLC39A1 and DRP1 by molecule docking. DRP1 (green); SLC39A1 (purple). (D) Co‐immunoprecipitation and co‐immunofluorescence showing interaction between SLC39A1 and DRP1 in MHCC97H cells. (E) Co‐immunoprecipitation showing interaction between MFF and DRP1 in SLC39A1 knockdown cells. (F) Cell proliferation determined by CCK8 assay in SLC39A1 silenced cells transfected with DRP1 overexpressed plasmid. (G, H) MMP level determined by TMRE and mitochondrial morphology determined by HSP60 immunofluorescent staining. (I) Western blot analysis of LC3 and LAMP1 in MHCC97H cells treated with pharmacological (Mdivi‐1) and genetic (siDRP1) inhibition of DRP1. Data are expressed as mean ± SD(n≥3). ***p < .001, **p < .01, and *p < .05.

Previous studies have indicated that SLC39A1 facilitates Parkin or Arih1‐mediated mitophagy by interacting with DRP1.^13^ Intriguingly, we found a positive correlation between the expression levels of SLC39A1 and DRP1 in LIHC datasets. In TCGA datasets, both DRP1 and SLC39A1 exhibited elevated expression in tumour tissues as opposed to normal tissues (Figure 5A). High DRP1 and SLC39A1 expressions were also observed in relapsed HCC clinical specimens compared with non‐relapsed specimens (Figure 5A; Table S1). Elevated DRP1 was correlated with poor overall survival in individuals with HCC (Figure 5B). Molecular docking displayed the physical interaction between DRP1 and SLC39A1 via forming hydrogen bonds through multiple interacting residues, including SER19 and LYS99, ALA16 and LYS99 (Figure 5C). Co‐immunoprecipitation and immunofluorescence staining verified the colocalization of SLC39A1‐DRP1 and the colocalization was reduced in SLC39A1‐silenced cells (Figure 5D). Previous efforts have demonstrated that mitochondrial fission factor (MFF) interacts with DRP1 on the mitochondrial outer membrane (MOM),^25^ but the absence of SLC39A1 does not affect the MFF‐DRP1 interaction (Figure 5E). Next, we determined whether cell proliferation was affected by the interaction between DRP1 and SLC39A1. Overexpression of DRP1 partially rescued the inhibition of cell proliferation in SLC39A1‐knockdown cells (Figure 5F). Subsequently, we hypothesized that the interaction of SLC39A1‐DRP1 is responsible for mitochondrial fission and MMP reduction. Pharmacological (Mdivi‐1) and genetic (siDRP1) inhibition of DRP1 potently reversed the inhibition of MMP in SLC39A1 overexpressed cells (Figure 5G; Figure S10A,B). Overexpression of DRP1 partially reversed the enhanced MMP in SLC39A1 silenced cells (Figure 5G; Figure S10B). Strikingly, inhibition of DRP1 significantly reduced mitochondrial fragmentation in SLC39A1 overexpressed cells (Figure 5H; Figure S10C). Similarly, overexpression of DRP1 reversed the mitochondrial fusion in SLC39A1‐silenced cell (Figure 5H; Figure S10D). Our findings indicated that the interaction of DRP1 and SLC39A1 contributes to MMP reduction, facilitating mitochondrial fission.

Herein, we found the mitochondrial calcium uniporter (MCU), a divalent ions transporter, interacted with SLC39A1 (Figure 5D; Figure S11A). Genetic inhibition of MCU abolished the inhibition of MMP in SLC39A1 overexpressed cells (Figure S11B,C). Knockdown of MCU largely rescued mitochondrial fragmentation induced by SLC39A1 (Figure S11D). These data indicated that MMP levels and mitochondrial fragmentation were regulated by SLC39A1 in an MCU‐dependent manner.

Next, to determine whether SLC39A1/DRP1/MCU axis contributes to the autophagic process, we treated cells with siDRP1 or siMCU and detected the expression levels of LC3 and Lamp1 in SLC39A1 overexpressed cells. Notably, the inhibition of DRP1 and MCU markedly attenuated LC3 and Lamp1 upregulation in SLC39A1 overexpressed cells (Figure 5I; Figure S11E), suggesting that the SLC39A1/DRP1/MCU axis plays a critical role in regulating autophagy and contributes to MMP reduction and mitochondrial fragmentation.

### 3.8 Genetic knockout of SLC39A1 attenuates DEN‐CCL4‐driven HCC development in mice

**FIGURE 6:**
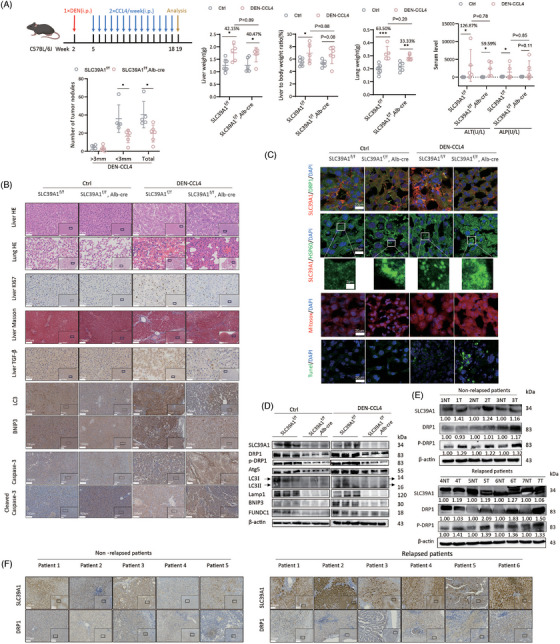
Liver‐specific knockout of SLC39A1 inhibits HCC development by repressing autophagy and promoting cell apoptosis in vivo. (A) Schematic illustration of DEN‐CCL4 model. Liver tumour nodules, liver weight, lung weight, liver to body weight ratio, serum levels of ALT and ALP in SLC39A1^f/f^,Alb‐Cre mice and SLC39A1^f/f^ mice (Ctrl: SLC39A1^f/f^ *n* = 7, SLC39A1^f/f^,Alb‐Cre *n* = 6; DEN‐CCL4: SLC39A1^f/f^ *n* = 5, SLC39A1^f/f^,Alb‐Cre *n* = 6). (B) Representative images of HE staining, Masson staining, immunohistochemical staining for Ki67, TGF‐β, LC3, BNIP3, Casp‐3 and Cleaved Casp‐3expressions in mice liver tissues (*n* = 5–7). (C) Representative images of immunofluorescent staining for DRP1, SLC39A1, HSP60, MitoSox Red staining and Tunel staining (*n* = 5–7). (D) Western blot analysis of intratumour DRP1, Atg5, LC3, Lamp1, BNIP3 and FUNDC1 expression in SLC39A1^f/f^,Alb‐Cre and SLC39A1^f/f^ mice (*n* = 3). (E) Western blot analysis of SLC39A1, DRP1 and P‐DRP1 expressions in liver tissues from non‐relapsed (*n* = 3) and relapsed (*n* = 4) HCC patients. NT: adjacent tissues; T: tumour tissues. (F) Representative images of immunohistochemical staining for SLC39A1 and DRP1 in tumour tissues from non‐relapsed (*n* = 5) and relapsed (*n* = 6) HCC patients. Data are expressed as mean ± SD. ***p < .001, **p < .01, and *p < .05.

We next crossed SLC39A1^f/f^ and Alb‐Cre mice to generate liver‐specific SLC39A1 knockout mice (SLC39A1^f/f^,Alb‐Cre) (Figure S12A). Here, using the diethylnitrosamine (DEN)‐carbon tetrachloride (CCl4) induced hepatocarcinogenesis mouse model (Figure 6A; Figure S12B), we found there was little effect of DEN‐CCl4 on body weight (Figure S12C) but markedly increased liver to body weight and lung weight in SLC39A1^f/f^ mice (Figure 6A). Of note, the increased magnitude of these weight indexes lessened in SLC39A1^f/f^,Alb‐Cre mice (Figure 6A). Liver‐specific deletion of SLC39A1 strongly reduced number of tumours in DEN‐CCL4 models compared with SLC39A1^f/f^ mice (Figure 6A). A modest reduction in serum alanine aminotransferase (ALT) and alkaline phosphatase (ALP) was noted in SLC39A1^f/f^,Alb‐Cre mice compared with SLC39A1^f/f^ mice with DEN‐CCL4 treatment (Figure 6A). Taken together, knockout of SLC39A1 suppressed liver tumorigenesis in mice.

### 3.9 Liver‐specific deletion of SLC39A1 represses autophagy and promotes apoptosis in mice

No noticeable substantial pathological change of liver and lung in both SLC39A1^f/f^,Alb‐Cre and SLC39A1^f/f^ mice without DEN‐CCL4 administration (Figure 6B). There were no discernible differences in terms of the presence of Ki67^+^ hepatocytes and level of transforming growth factor‐beta (TGF‐β) regardless of untreated SLC39A1^f/f^,Alb‐Cre or untreated SLC39A1^f/f^ mice (Figure 6B). We assessed the histological characteristics of the liver and identified widespread cellular necrosis, notable for eosinophilic cytoplasmic changes and cellular swelling, accompanied by obvious necrosis lesions of the lung in DEN‐CCL4 models regardless of SLC39A1^f/f^,Alb‐Cre or SLC39A1^f/f^ mice (Figure 6B). Ki67^+^ hepatocytes, TGF‐β and fibrosis of surrounding hepatocytes were markedly increased in both SLC39A1^f/f^,Alb‐Cre and SLC39A1^f/f^ mice injected with DEN‐CCL4. However, liver‐specific SLC39A1 deletion alleviated above pathological phenotypes, reversed increased Ki67^+^ hepatocyte proliferation, decreased TGF‐β positive hepatocytes and significantly reduced fibrosis in surrounding hepatocytes compared with SLC39A1^f/f^ mice in DEN‐CCL4 models (Figure 6B).

Apparently, liver‐specific SLC39A1 deletion led to a reduction of the colocalization area of SLC39A1‐DRP1 (Figure 6C). Consistent with the in vitro results, liver‐specific SLC39A1 deletion significantly suppressed autophagy and mitophagy evidenced by reduced LC3, Lamp1, BNIP3 and FUNDC1 regardless of DEN‐CCL4 treatment (Figure 6D; Figure S12D). Notably, liver‐specific SLC39A1 deletion induced mitochondrial aggregation, consistent with a marked downregulated of DRP1 (Figure 6C; Figure S12D). Enhanced ROS accumulation and hepatocyte apoptosis were observed in SLC39A1^f/f^,Alb‐Cre mice compared with SLC39A1^f/f^ mice after DEN‐CCL4 injection, accompanied by increased cleaved Casp‐3 (Figure 6B,C).

### 3.10 SLC39A1 is highly expressed in recurrent HCC patients

SLC39A1 was differentially expressed between relapsed and non‐replaced HCC patients by RNA sequencing using our in‐house HCC cohort (Table S1), and high SLC39A1 is associated with poor survival by analyzing the TCGA dataset. To further validate this observation, we collected liver adjacent tissues (NT) and tumour tissues (T) from 5 non‐relapsed and 6 relapsed HCC patients after surgical resection (Table S2). Intriguingly, compared with adjacent tissues, SLC39A1 expression was significantly higher in the tumour tissues no matter in nonrelapsed or relapsed HCC patients (Figure 6E; Figure S12E). More importantly, liver tissues from relapsed patients presented higher levels of SLC39A1 than non‐relapsed patients, regardless of adjacent or tumour tissue (Figure 6E; Figure S12E). Consistently, the expression levels of DRP1 and p‐DRP1 were both higher in tumour tissues than adjacent liver tissue in relapsed HCC patients. In non‐relapsed HCC patients, only the p‐DRP1 level, but not the total DRP1 level, was significantly higher in tumour tissues than adjacent liver tissues. Most importantly, compared with adjacent tissues, tumour tissues from relapsed patients presented higher levels of DRP1 and p‐DRP1 than non‐relapsed patients (Figure 6E; Figure S12E). Immunohistochemical staining also showed increased SLC39A1 and DRP1 in relapsed HCC samples compared with non‐relapsed HCC samples (Figure 6F). Thus, high SLC39A1 and DRP1 levels are associated with HCC recurrence.

### 3.11 Specific peptide targeting SLC39A1 inhibits cell proliferation and tumour growth

**FIGURE 7:**
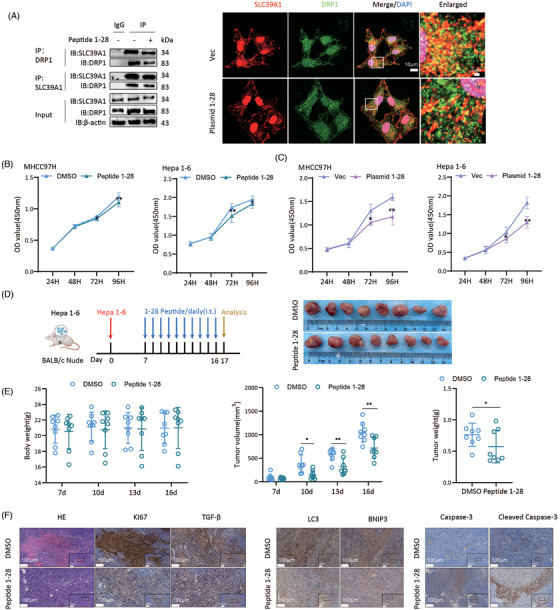
Specific peptide targeting SLC39A1 attenuates HCC progression in vivo and in vitro. (A) Co‐immunoprecipitation and co‐immunofluorescence analysis of interaction between SLC39A1 and DRP1 in MHCC97H cells treated with or without specific peptide (10 µM) and plasmid targeting SLC39A1. (B, C) Cell proliferation analysis in MHCC97H and Hepa1‐6 cells treated with specific plasmid or peptide (10 µM) targeting SLC39A1. (D) Tumour volumes and cell proliferation in mice by subcutaneous injection with Hepa1‐6 cells followed by treatment with DMSO or specific peptide targeting SLC39A1 (2.5 mg/g) through intratumoral injection daily for 10 days (*n* = 8). (E) Tumours were harvested on day 17 and dissociated for tumour volume and tumour weight examination (*n* = 8). (F) Representative images of HE staining, and immunohistochemical staining for Ki67, TGF‐β, LC3, BNIP3 and cleaved Casp‐3 (*n* = 8). Data are expressed as mean ± SD. ***p < .001, **p < .01, and *p < .05.

A region containing 28 hydrophilic amino‐acid at the N terminus of SLC39A1 was documented as the primary region to interact with DRP1.^13^ We designed specific plasmid and peptide targeting SLC39A1 to interfere with the interaction of SLC39A1 and DRP1. Co‐immunofluorescence staining verified the reduced colocalization of SLC39A1‐DRP1 in MHCC97H cells treated with a specific plasmid targeting SLC39A1 (Figure 7A; Figure S13A). Importantly, specific plasmid and peptide targeting SLC39A1 significantly reduced the proliferative capability of MHCC97H and Hepa1‐6 cells (Figure 7B,C; Figure S13B). We then investigated the anti‐tumour effect of SLC39A1 specific peptide in vivo. Both the tumour volume and tumour weight were markedly decreased in mice with peptide treatment (Figure 7D,E; Figure S13C).

Mice treated with specific peptide targeting SLC39A1 showed attenuated disseminated cell necrosis and necrosis lesions and markedly decreased Ki67^+^ hepatocytes and TGF‐β compared with DMSO (Figure 6F). Decreased LC3 and BNIP3, and increased Cleaved Casp‐3 were observed in mice with specific peptide treatment (Figure 6F). These results indicated specific peptide targeting SLC39A1 by interfering combination of full‐length SLC39A1 and DRP1 inhibits cell proliferation and tumour growth, which offers a promising novel strategy for HCC treatment.

## 4 DISCUSSION

The HCC incidence and mortality rates for male gender are two to three times higher compared with female, both worldwide and in China.^1^, ^26^ There are 20% (343/1716) in female and 80% (1373/1716) in male of HCC incidence rates.^27^ In our cohort, liver specimens from patients undergoing curative surgical resection were continuously collected without intentionally pursuit the ratio of male or female. HCC incidence rates of male patients (81.5%, 22/27) in our study were consistent with the above‐reported ratio.

Our transwell experiment demonstrated that SLC39A1 deletion significantly reduced the invasion and migration in vitro, while the in vivo SLC39A1 expression levels are no significant difference between metastatic and nonmetastatic HCC patients. This suggests that the effects of SLC39A1 on cell migration in vitro may not fully translate to its role in promoting metastasis of HCC in vivo. The discrepancies may be attributed to differences in the microenvironment, gene regulation, cellular heterogeneity, immune system involvement, metabolic states, limitations of experimental models, and context‐dependent gene functions. For example, tumours exhibit high cellular heterogeneity, and different subpopulations of cells may respond differently to the gene's function. To comprehensively understand the role of a gene in tumour metastasis, it is essential to integrate findings from multiple experimental models and clinical data.

We found that SLC39A1 promotes cell proliferation and migration, but inhibits cell apoptosis in HCC. Consistent with our findings, a previous report analyzing samples from the Oncomine database suggested that high SLC39A1 is linked to the progressed TNM stage, and poor clinical outcome in HCC.^28^ Additionally, it facilitates oncogenesis in HCC.^28^ SLC39A1 is negatively correlated with NK cell activation and monocytes in gliomas and B‐cell immunity in Pan‐Cancer, contributing to tumour immune evasion and NK cell‐mediated cytotoxicity suppression.^29^ GSEA demonstrated that SLC39A1 overexpression is associated with carcinogenesis‐related pathways, including the Wnt/MAPK signalling pathway, cell cycle, NOD‐like receptor and TGF‐β signalling pathway, evidenced by increased cyclin D1, MMP2, among which Cyclin D and cell cycle were also downregulated in shSLC39A1 cells in our study. Apart from regulating tumour cells, elevated SLC39A1 expression is linked to the enhanced presence of Th2 cells but reduced infiltration of cytotoxic cells, contributing to adverse clinical prognoses in HCC.^28^ Interestingly, diminished intracellular zinc levels influence the Th1/Th2 cytokine equilibrium and activation of immune cell populations.^30^, ^31^ Therefore, we speculate that SLC39A1 facilitates cytotoxic T cells and reduces Th2 cell infiltration by elevating cellular Zn^2+^ levels, which finally forms a tumour‐promoting microenvironment. This also explained our finding that Zn^2+^ is not involved in SLC39A1‐regulated pro‐tumour activity in hepatocytes. On the contrary, a study using an early‐stage hepatocellular carcinoma (EHCC) cohort indicates that tumour tissues exhibit decreased expression of SLC39A1 as compared with the adjacent tissues.^32^ In this study, the expression of SLC39A1 is evaluated by intensity score by calculating the percentage of positive cells by immunohistochemistry. The highly subjective and poorly accurate immunohistochemistry assessment might contribute to the observed decrease of SLC39A1 in EHCC, which is contradicted by ours’ and most other studies. Alternatively, it is possible that the feature of early‐stage HCC largely differs from intermediate and advanced HCC. SLC39A1 is also reported to suppress tumour growth in prostate cancer by inhibiting NF‐κB and Ras pathways, leading to decreased levels of apoptotic‐inhibitory factors Bcl‐2/XL and tumorigenic cytokines IL‐6/8.^33^

In our study, MMP levels and mitochondrial fragmentation are regulated by SLC39A1 in a DRP1/MCU‐dependent manner. Intriguingly, reciprocal regulation between the SLC39A1‐DRP1 complex and MMP level can establish a feedback mechanism: DRP1 recruitment induced by SLC39A1 can initiate MMP imbalance and mitochondrial fragmentation, subsequently inducing further DRP1 recruitment. This process facilitates the isolation of impaired mitochondrial segments from the mitochondrial network, facilitating their targeted degradation. The outcome for fragmented mitochondria is governed by their capability to recover MMP balance.^34^ Interestingly, functional mitochondria can rejuvenate MMP balance and continue to participate in the fusion process within the mitochondrial network, whereas those unable to restore MMP stability are eliminated through mitophagy.^35^ SLC39A1‐DRP1 axis could be responsible for the upregulation of the cellular autophagic flux.

Aberrant expression of DRP1 is intricately involved in a variety of diseases. DRP1‐mediated mitochondrial fission presented in HCC promotes tumour‐associated macrophage infiltration by triggering intracellular mtDNA stress and increasing CCL2 release through the TLR9‐dependent NF‐κB signalling pathway.^36^ Alcohol consumption decreases hepatic DRP1 in mouse models, leading to impaired mitophagy and dysfunctional innate immune response, which ultimately aggravates liver injury in alcohol‐associated liver disease.^37^ Studies also reported the beneficial effect of DRP1 in non‐alcoholic steatohepatitis (NASH). DRP1 alleviates NASH by reducing ER stress, inhibiting Oma1 activation and integrated stress response.^38^ Inherent DRP1 protects the mice heart from high‐fat diet‐induced obesity cardiomyopathy by facilitating mitophagy.^39^ The expression of DRP1 is significantly increased and enhances the proliferation and regenerative capacity in muscle stem cells.^40^ These findings highlight unbalanced mitochondrial dynamics mediated by DRP1 in a variety of diseases.

Both our and previous studies demonstrated autophagy provides an important cell survival mechanism by preventing ROS accumulation. ROS acts pro‐tumorigenic activity by augmenting NRF2, increasing Ca^2+^ and stimulating mitochondrial fission, which in turn facilitates autophagy through NF‐κB‐mediated upregulation of p62/SQSTM1.^41^ On the contrary, ROS impair fatty acid oxidation, leading to a reduction of cellular ERK and DRP1, thereby suppressing mitochondrial fragmentation and inhibiting cell migration via GCN5L1 in HCC.^42^ Tumour cells elevated antioxidant capacity to regulate ROS‐mediated growth and avoid ROS thresholds to trigger apoptosis or ferroptosis.^43^ Interestingly, increased ROS also activates TP53, which suppresses cancer growth by regulating genes associated with cell cycle arrest and apoptosis.^44^ Collectively, ROS influence cancer development through seemingly opposing mechanisms: either promoting tumour growth and aiding cancer cell conversion and multiplication or inducing cell death by various pathways.^45^

MCU is not only a transporter for Zn^2+^ but also for Ca^2+^, which is widely reported to regulate mitochondrial homeostasis. Indeed, the MCU complex, along with its regulatory components MICU1, MICU2, and EMRE, ensures precise control of mitochondrial calcium uptake to maintain cellular homeostasis by regulating MMP and mitochondrial dynamics. Silence of MCU is widely known to reduce mitochondrial calcium uptake and increase cytosolic calcium levels, thereby triggering DRP1‐mediated mitochondrial fission.^46^ However, MCU knockdown is also reported to counterintuitively reduce intracellular free calcium levels and substantially inhibit the translocation of DRP1 and its interaction with Fis1 receptors, resulting in decreased mitochondrial fission.^47^ Similarly, homocysteine activation of MCU was also found to increase mitochondrial fission by enhancing calcium transferring into mitochondria.^48^ These studies collectively indicate that the regulation of mitochondrial dynamics by MCU exhibits complexity and context‐dependent specificity, primarily attributable to its differential modulation of cytoplasmic calcium signalling across distinct physiological conditions.

Our data highlight the essential function of SLC39A1‐DRP1 interaction in mitochondrial quality control in HCC, which accelerates autophagic flux and contributes to the occurrence and recurrence of HCC. Specifically, our findings have indicated the presence of a complex of SLC39A1 with DRP1, MCU and MFF, where these proteins collaborate to recruit more cargoes to affect mitochondrial quality control. Given above proteins are involved in the wide range of pathways in HCC, activation of autophagy along with mitochondrial fission and the decreased ROS levels could be the underlying mechanisms for the HCC proliferation and recurrence mediated by the interaction of SLC39A1‐DRP1. The current study did not explore the precise molecular mechanisms underlying the regulatory relationship between SLC39A1 and DRP1. Future investigations are required to elucidate whether this regulation occurs at transcriptional or post‐translational levels and to identify potential intermediary signalling pathways or protein interactions involved in this process.

The use of peptides in anti‐tumour therapy can enhance target engagement and drug penetration in the in vivo tumour microenvironment, potentially activate the immune system, and increase drug stability and accumulation via in vivo circulation. Of note, targeting SLC39A1 by specific peptides efficiently suppresses HCC proliferation, which opens an avenue for intervention and reduces the recurrence rate after LR patient removal.

## AUTHOR CONTRIBUTIONS

Rui Li and Zhe Wang: Data curation, writing—original draft, formal analysis, statistical analysis, writing—review and editing. Lixin Cheng and Qiong Wu: Data curation, investigation, writing—review and editing. Zhiqiang Cheng: Methodology. Dong Ji: Data curation, resources, investigation, and methodology. Fengjuan Chen and Qingxian Cai: Conceptualization and funding acquisition. Yijin Wang: Conceptualization, resources, supervision, funding acquisition, methodology, writing–original draft, writing–review and editing, and project administration.

## CONFLICT OF INTEREST STATEMENT

The authors declare no conflict of interest.

## ETHICS STATEMENT

This study involving human tissues was performed in accordance with the Declaration of Helsinki and approved by the Ethics Committee of Fifth Medical Center of Chinese PLA General Hospital (license number: 2020055D, 14 July 2020) and the Third People's Hospital of Shenzhen (license number: 2024–181, 9 July 2024). All participants signed written informed consent. The animal experiments were performed with international guidelines of IACUC and the approval of the Institutional Animal Care and Use Committee of Southern University of Science and Technology (license number: JY202304004; 26 April 2023).

## Supporting information



Supporting information

## Data Availability

All data generated or analysed during this study are included in this article. The data from the RNA sequencing analysis used in this research can be found in the National Genomics Data Center (PRJCA004993) and the GEO database (GSE268875).
