# Supplementary material for: Coordination of SLC39A1 and DRP1 facilitates HCC recurrence by impairing mitochondrial quality control
Source: Clin Transl Med. 2025 Jun 3;15(5):e70362. doi: 10.1002/ctm2.70362 (PMC12134400; doi:10.1002/ctm2.70362)
Supplement: Supplementary file 1 — Supporting information [file CTM2-15-e70362-s001.docx]

**Materials and methods**

**RNA-sequencing**

HCC samples from 12 non-relapsed and 15 relapsed HCC patients who underwent surgically resection at Fifth Medical Center of Chinese PLA General Hospital from June 2012 to April 2016 were continuously collected for RNA sequencing. RNA sequencing transcriptomics was performed to identify downstream pathways between SLC39A1-knockdown cells and control cells. RNA was extracted by RNeasy FFPE Kit (Qiagen Inc., Germantown, USA). RNA library was constructed with TruSeq RNA Exome kit (Illumina) and HiSeq X10 platform (Illumina). Sequencing reads containing many Ns (> 5%), low-quality bases (> 15% bases with quality ≤ 19) and adaptor sequences were discarded. Then paired-end reads with high-quality were retained for further data analysis. High-quality reads were mapped to the reference genome hg19 via Bowtie software (version 2.2.4) with default parameters. Gene expression was calculated as reads per kilobase per million reads (FPKM) with Cufflinks software (version 2.2.1, default parameters).

**Analysis of DEGs Gene enrichment analysis**

To identify the DEGs, an R language script was made, and a limma package (<http://www.bioconductor.org/packages/release/bioc>/html/limma.html) on 2 January 2020 was downloaded and performed by the R software. The false discovery rate (FDR)<0.05 and fold change>1.5 were set as the cutoff values to identify the DEGs. The DEGs were presented using the volcano plot script by R software. The DEGs were subjected to clusterProfiler package in R software for GO, KEGG and GSEA pathway enrichment analysis to determine their potential functions and pathways.

**Immune infiltration analysis**

Estimation of Immune cells in Malignant tumor tissues using Expression data (CIBERSORT) is a method that uses gene expression matrix to infer the proportion of immune cells in tumor samples. We use the “CIBERSORT” package to calculate the immune cell proportion. The correlation between immune cells proportion and the expression of SLC39A1, SLC50A1 and SLC66A3 were explored through Spearman Correlation Analysis.

**Lentivirus packaging and Infection**

Lentiviral pLKO.1-shRNA plasmids targeting SLC39A1, SLC50A1 and SLC66A3 were constructed. For lentivirus packaging, pLKO.1-control, pLKO.1-shSLC39A1, pLKO.1-shSLC50A1 and pLKO.1-shSLC66A3 were transfected into HEK-293T cells using a co-transfection system (pMD2G: psPAX2: target plasmids=1:3:4) with PEI (Yeasen). The shRNA sequences were designed by Sangon Biotech. The culture supernatant was collected at 48 and 72 h after transfection and then filtered through a 450 nm filter. When cells reached approximately 30% coverage in 6-well plates, 0.5ml of lentivirus, 0.5 ml of fresh medium were added for infection. After incubation for 24 h, puromycin (2 µg/µl) was used for three days to select cells that were successfully infected. All sequences used in the manuscript are provided in the Supplementary Table 3.

**RNA extraction and quantitative real‑time PCR (RT‑qPCR)**

Cells plated with 12-well culture plates for RNA extraction. Liver samples of mice were collected and stored at -80℃. Chloroform-free RNA extraction kit® (BioTeke) was used to extract total RNA from tissues and cells at 4 °C, followed by quantitative real-time PCR (RT-qPCR). RNA was reverse-transcribed using the HiScript II Q RT SuperMix for qPCR® (Vazyme) to create cDNA, which was then amplified using the Hieff UNICON® Universal Blue qPCR SYBR Green Master Mix (Yeasen). The expression of the amplification products was calculated using the delta-delta comparative (2^-ΔΔCT^) approach. All of the experiments were done at least three times. All primers used in the manuscript are provided in the Supplementary Table 3. The primers were purchased from Sangon Biotech.

**Western blot analysis**

Cells plated with 12-well culture plates for Western blot. Liver samples were collected and and stored at -80℃. Harvested cells or liver samples were lysed in RIPA lysis buffer (APExBIO) supplemented with a mixture of PMSF (Beyotime). Following centrifugation at 12,000 rpm for 10 min at 4°C, supernatant was collected as the whole-cell lysate. Protein concentrations of the whole-cell lysates were determined using Nanodrop (Thermo Fisher). Western blot analysis was performed as equal amounts of cell lysate (40 µg) were subjected to 8%-12% sodium dodecyl sulfate-polyacrylamide gel electrophoresis and transferred onto polyvinylidene fluoride membranes (Bio-Rad Laboratories) using the Bio-Rad electro-transfer system (Bio-Rad Laboratories). The membranes were blocked with 5% non-fat milk in TBST (20 mM Tris-HCl, 150 mM NaCl, and 0.1% Tween-20) at room temperature for 1-2 h and probed with specific primary antibodies (1:1000-1:2000) overnight at 4℃. The membranes were then washed thrice with TBST and incubated with HRP-conjugated secondary antibodies (1:5000) in TBST at room temperature for 1 h. The specific protein bands were visualized using custom-made ECL (Sinsage) detection system. All antibodies used in the manuscript are provided in the Supplementary Table 3.

**Assessment of cell proliferation**

Cells plated with 96-well culture plates for CCK-8. The cellular proliferative rates were determined using a Cell Counting Kit-8® (CCK-8, Yeasen). 100 µl of cell suspension with a density of 3×10^4^/ml was added to each well of a 96-well plate, and 10 µl of CCK-8 reagent was added at the indicated times. The absorbance at 450 nm was measured to evaluate cell proliferation after 1 h of incubation.

HCC cells were seeded into six-well plates. A colony formation assay was used to measure the capacity of cells to proliferate. After 14 days of culture, the cells were fixed and Crystal Violet stained, and the total colonies formed were counted.

**Cell invasion and migration assays**

Cells plated with 24-well culture plates for Transwell assay. Cell invasion tests were carried out in 8-µm Transwell chambers. HCC cells were resuspended in FBS-free basal media and inoculated on the upper chamber pre-plated with 100 µl of diluted Matrigel (Yeasen). The bottom chamber was filled with an FBS-enriched complete medium. After 36h, 72h of culture, the chamber was fixed with 4% paraformaldehyde and stained with 0.5% crystal violet. The crystal violet was then rinsed away, and the cells were counted using a microscope. The migration experiment was similar to the invasion experiment, with the exception that Matrigel was not utilized to cover the upper chamber. After being fixed and dyed, the migrated cells were viewed under a microscope.

In wound-healing assays, cells were initially seeded in 6-well plates. When the cells reached approximately 80% confluence, sterile 200μl pipette tips were gently used to create scratches in the cell monolayer. Subsequently, these treated cells were incubated for an additional 48 h in serum-free culture medium. The healing process of cell injuries was observed using an inverted microscope produced by Mshot.

**Flow cytometry analysis**

In this study, flow cytometry was used to detect cell apoptosis, cell cycle and Zn^2+^ levels. The cells (1×10^5^) were incubated with Annexin V-FITC/PI and Zinpyr-1 according to the manufacturer’s protocols. Labeled cells were washed with staining buffer twice and analyzed by BD FACSCantoII Analyzer (BD Biosciences). The cytometry results were quantified by FlowJo software (BD Biosciences).

**Immunohistochemistry (IHC)**

Tissues were fixed in 10% neutral buffered formalin, embedded in paraffin, and cut into 5 μm sections and used for IHC staining with specific primary antibodies. The sections of formalin-ﬁxed and parafﬁn-embedded tumor tissues were dewaxed and subjected to heat-mediate antigen retrieval using EDTA antigen retrieval solution (Beyotime). After incubation of 3% H_2_O_2_ to inactivate endogenous peroxidase for 15 min and 10% normal goat serum to block nonspeciﬁc sites for 30 min, and then were incubated with the primary antibody at 4 ℃ overnight. After rinsing with Tris-buffered saline, the slides were incubated for 45 min with biotin-conjugated secondary antibody, washed, and then incubated with enzyme conjugate horseradish peroxidase (HRP)-streptavidin. Freshly prepared DAB (Gene Tech) was used as a substrate to detect HRP. Finally, slides were counter-stained with hematoxylin and mounted with aqueous mounting media.

**Hematoxylin and eosin (HE) staining**

After airdrying, the paraffin sections were baked at 60℃-62℃ for 20 min, washed with xylene for 5 min 3 times, soaked in graded alcohols (100%×2, 95%×1 and 80%×1) for 3 min each, and ﬁnally rinsed in tap water for 5 min. They were then stained with hematoxylin for 5 min and rinsed in tap water for 30 s, followed by brief submersion in an acid solution and 30-90 s in ammonia water. They were then washed with water for 10 min, 80% ethanol for 1 min, counterstained with eosin for 1 min, dehydrated in graded alcohols followed by xylene as above, and mounted with aqueous mounting media.

**Masson staining**

The Masson stain processed according to Servicebio technology. The paraffin sections were immersed in sequence in environmentally friendly dewaxing transparent liquid I for 20min; environmentally friendly dewaxing transparent liquid II for 20min; anhydrous ethanol I for 5min; anhydrous ethanol II for 5min; 75% ethyl alcohol for 5min, and then rinsed with tap water. The frozen sections were removed from the -20℃ refrigerator and restored to room temperature, fixed with tissue fixating solution for 15min, and then rinsed with running water. Then the slices were soaked in Masson A overnight, rinse with tap water. Masson B and Masson C were prepared into Masson solution according to the ratio of 1:1. Then stain with Masson solution for 1 min, rinse with tap water. Differentiate with 1% hydrochloric acid alcohol for several seconds, rinse with tap water. Soak the slices in Masson D for 6 min, rinse with tap water. Masson E for 1 min, slightly drain directly into Masson F for 2-30s. Rinse the slices with 1% glacial acetic acid and then dehydration with two cups of anhydrous ethanol. Clearing and sealing: slides were soaked in 100% ethanol for 5 min; Xylene for 5 min; finally sealed with neutral gum.

**Immunofluorescence (IF)**

All cells used were seeded on cover slides in 12-well plates, incubated overnight and then fixed in 4% paraformaldehyde for 20 min, permeabilized with 0.5% Triton X-100 for 5 min, blocked in 1% normal goat serum for 60 min, and incubated with primary antibodies for overnight at 4℃, then washed thrice with TBST, followed by secondary antibodies for 60 min at RT. Nuclei were stained with 4′,6-diamidino-2-phenylindole dihydrochloride (DAPI) at RT for 10 min. Photographs were captured with a laser confocal microscopy (Zeiss). And the tissue apoptosis analysis processed according to the manufacturer instructions of Servicebio® Fluorescein (FITC) Tunel Cell Apoptosis Detection Kit (Servicebio).

**Zinc concentration regulation and staining.**

ZnCl_2_ and TPEN were used to increase and reduce intracellular Zn^2+^, respectively. The fluorescent reporter Zinpyr-1 (APExBIO) was used to detect labile Zn^2+^ by Flow cytometry (BD Bioscience) and Confocal microscopy (Zeiss).

**Determination of mitochondrial reactive oxygen species (ROS) generation**

MitoSOX Red fluorescent probe was used to determine the mitochondrial ROS production. MitoSOX Red reagent (MedChemExpress), a live-cell permeant, selectively targets mitochondria and exhibits red fluorescence when oxidized by superoxide. Briefly, 5×10^4^ HCC cells were seeded per well in twelve-chamber slides. After the treatment period, cells were washed with PBS and incubated with 2.5 μM MitoSOX Red for 30 min at 37℃ in 5% CO_2_. Then, the cells were imaged using a fluorescence microscope (Zeiss).

**Mitochondrial membrane potential (ΔΨ m) assay**

MHCC97H cells were seeded into 12-well plates at 5×10^4^ cells/well, and incubated overnight. At each time, cells in the positive control group were treated with 10 µM carbonyl cyanide-m-chloro phenylhydrazone (CCCP) for 10 min. Subsequently, all cells were treated with tetramethylrhodamine, ethyl ester (TMRE) for 30 min and the nuclei stained with DAPI for 30 min. Cells were immediately observed under a fluorescence microscope and photographed.

**Isolation of mitochondria**

Mitochondria were isolated using a mitochondria isolation kit (Beyotime). Briefly, 2-5×10^7^cells were washed twice with precooling 1×PBS and lysed in mitochondrial separation reagents with PMSF, incubated on ice for 10-15 min, followed by grinding and gradient centrifugation.

**Co-immunoprecipitation**

Cells were lysed in IP lysis buffer (Beyotime) containing a proteinase and phosphatase inhibitor cocktail (Selleck). The cell lysates were incubated with antibodies in a rotating incubator overnight at 4 °C, following the co-incubation with protein A/G magnetic beads (Beyotime) at 4 °C for 6 h. Beads were washed with PBST and boiled in 1×SDS-loading buffer (Beyotime) and the binding proteins were analyzed by Western blot analysis.

**Molecular docking**

The predicted structures of SLC39A1 and DRP1 were generated by Alphafold. To ensure the accuracy of the docking results, the protein was prepared by the AutoDockTools-1.5.7, and the water molecules were manually eliminated from the protein and the polar hydrogen was added. Docking Web Server (GRAMM) was used for protein-protein docking. The resulting protein-protein complex was also manually optimized by removing water and adding polar hydrogen by the AutoDockTools-1.5.7. Finally, the protein-protein interactions were predicted and the protein-protein interaction figure was generated by PyMOL.

**Statistical analysis**

Statistical analyses were performed using GraphPad Prism, version 8.0. Significance between two groups was analyzed using a two-tailed unpaired or paired Student’s t test or one-way ANOVA. The significance of clinical features was assessed by Fisher exact test or the U test. Prognostic analysis was performed using the Kaplan-Meier method and log rank test. N=3 independent experiments unless stated otherwise. The data are presented as the means ± SD, unless indicated otherwise. Differences were considered statistically significant when P < 0.05 (*), P < 0.01 (**) or P < 0.001 (***).

**Supplementary Table and Figure**

**Supplementary Table 1. Comparison of baseline characteristics, complications and laboratory parameters between 12 non-relapsed and 15 relapsed HCC patients after surgically resection.** **HCC samples were collected for RNA sequencing.**

| Variable | Non-Relapsed  N = 12 | | Relapsed N = 15 | *P* |
| --- | --- | --- | --- | --- |
| **Age(year)** | 52 (42-57) | | 50 (44-58) | 0.934 |
| **Gender** |  | |  |  |
| Male | 10(83.33%) | | 12(80%) |  |
| Female | 2(16.67%) | | 3(20%) |  |
| **Tumor thrombus** | 5(41.67%) | | 11(73.33%) | 0.103 |
| **DFS (weeks)** | 312.9±18.87 | | 14.47±13.13 | <0.001 |
| **Laboratory parameters** |  | |  |  |
| **Liver function** |  | |  |  |
| Total protein (TP) 60-83 (g/L) | 69.33±3.89 | | 65±5.17 | 0.024 |
| Albumin (ALB) 35-55 (g/L) | 43(37.5-44) | | 39(37-42) | 0.193 |
| Globulin (GLOB) 20-40 (g/L) | 28.5(24.25-30.75) | | 24(23-28) | 0.059 |
| Albumin/Globulin 1.2-2.4 | 1.51(1.34-1.71) | | 1.66(1.46-1.76) | 0.516 |
| Alanine aminotransferase (ALT) 5-40 (U/L) | 28±12.26 | | 47.93±60.93 | 0.277 |
| Aspartate aminotransferase (AST) 5-40 (U/L) | 26.08±3.87 | | 42.53±39.36 | 0.163 |
| Alkaline phosphatase (ALP) 40-150 (U/L) | 69.92±15.43 | | 112.1±69.77 | 0.051 |
| Cholinesterase (CHE) 5000-12000 (U/L) | 7108(7037-8000) | | 6288(4842-7176) | 0.006 |
| Total bilirubin (TBIL) 3.4-20.5 (umol/L) | 15.15±5.13 | | 64.99±110.8 | 0.134 |
| Direct bilirubin (DBIL) 0-6.8 (umol/L) | 5.8±2.17 | 45.91±89.23 | | 0.134 |
| Creatinine (CRE) 53-97 (umol/L) | 84(70-94) | | 76(67-83) | 0.134 |
| Blood urea nitrogen (BUN) 2.9-7.5(mmol/L) | 5.1(3.92-5.72) | | 4(3.5-5.6) | 0.461 |
| **Serum tumor markers** |  | |  |  |
| Alpha fetoprotein (AFP) 0-10.0 (ng/ml) | 61.97±112.9 | | 581.6±909.9 | 0.061 |
| **Hematological examination** |  | |  |  |
| Leukocyte count (WBC)3.97-9.15 (10^9^/L) | 4.83(4.24-7.49) | | 4.67 (3.55-6.07) | 0.48 |
| Neutrophilic granulocyte percent (NEU%) 50-70 (%) | 0.57(0.44-0.65) | | 0.65 (0.57-0.72) | 0.118 |
| Neutrophilic granulocyte count (NEUT) 2-7 (10^9^/L) | 2.46(2.01-4.58) | | 2.78 (1.87-4.35) | 0.857 |
| Lymphocyte percent (LYMPH%) 20-40 (%) | 0.32(0.24-0.39) | | 0.26 (0.16-0.31) | 0.132 |
| Lymphocyte count (LYMN) 0.8-4.0 (10^9^/L) | 1.51±0.81 | | 1.24±0.61 | 0.342 |
| Neutrophil/Lymphocyte Ratio | 1.79(1.14-2.69) | | 2.53 (1.87-4.35) | 0.909 |
| Monocytes count (MIDN) 0.12-1.0 (10^9^/L) | 0.46±0.17 | | 0.34±0.8 | 0.101 |
| Erythrocyte count 0-1 (10^9^/L) | 4.56(4.07-5.02) | | 4.51 (4.16-4.78) | 0.519 |
| Hemoglobin (HB) 131-172 (g/L) | 152.5(136.8-161.8) | | 144(135-154) | 0.209 |
| Platelet count (PLT) 85-303 (10^9^/L) | 131.3±43.4 | | 147.1±111.6 | 0.648 |
| Prothrombin activity (PTA)75-100 (%) | 92.69±8.93 | | 87.19±8.52 | 0.115 |
| Prothrombin time (PT)10-14(s) | 11.7(10.62-12.15) | | 12 (11.6-12.5) | 0.398 |
| Thrombocytocrit (PCT) 0.06-0.40 (%) | 0.21±0.19 | | 0.21±0.18 | 0.953 |

^*^ Continuous variables were expressed in mean ± standard deviation (SD) or median (interquartile range [IQR]). Categorical variables were presented as number (percentage).

**Supplementary Table 2. Comparison of baseline characteristics, complications and laboratory parameters between 5 non-relapsed and 6 relapsed HCC patients after surgically resection. The adjacent tissues and tumor tissues from HCC patients were used for protein expression and histological staining.**

| Variable | Non-Relapsed  N = 5 | Relapsed N = 6 | *P* |
| --- | --- | --- | --- |
| **Age(year)** | 38 (32.5-58) | 49 (42-56.25) | 0.483 |
| **Gender** |  |  |  |
| Male | 5(100%) | 6(100%) |  |
| Female | 0(0%) | 0(0%) |  |
| **Tumor thrombus** | 0(0%) | 4(66.67%) | 0.019 |
| **DFS (weeks)** | 137.5±26.1 | 48.45±57.83 | 0.012 |
| **Laboratory parameters** |  |  |  |
| **Liver function** |  |  |  |
| Total protein (TP) 60-83 (g/L) | 74.6(66.4-78.55) | 68.225(62.68-72.78) | 0.195 |
| Albumin (ALB) 35-55 (g/L) | 44.8(42.25-49.4) | 40.15(38.6-44.25) | 0.066 |
| Globulin (GLOB) 20-40 (g/L) | 27.5(23.05-31.4) | 25.5(23.23-31.08) | 0.862 |
| Albumin/Globulin 1.2-2.4 | 1.55(1.49-1.99) | 1.62(1.27-1.82) | 0.442 |
| Alanine aminotransferase (ALT) 5-40 (U/L) | 29(22.5-52.5) | 28(22-41.5) | 0.568 |
| Aspartate aminotransferase (AST) 5-40 (U/L) | 21(18.5-35.5) | 27.5(22.75-34.25) | 0.671 |
| Alkaline phosphatase (ALP) 40-150 (U/L) | 76(62-94) | 88.5(80-96.5) | 0.255 |
| Cholinesterase (CHE) 5000-12000 (U/L) | 8584(8273-10708) | 7577(5266-8252) | 0.039 |
| Total bilirubin (TBIL) 3.4-20.5 (umol/L) | 13.34±3.72 | 13.65±10.13 | 0.95 |
| Direct bilirubin (DBIL) 0-6.8 (umol/L) | 4.44±1.14 | 4.41±2.81 | 0.987 |
| Creatinine (CRE) 53-97 (umol/L) | 90(75.5-100.5) | 86(69-90.9) | 0.468 |
| **Serum tumor markers** |  |  |  |
| Alpha fetoprotein (AFP) 0-10.0 (ng/ml) | 1021±2256 | 12949±31668 | 0.426 |
| **Hematological examination** |  |  |  |
| Leukocyte count (WBC)3.97-9.15 (10^9^/L) | 6.62(4.03-7.45) | 5.53(4.79-6.42) | 0.769 |
| Neutrophilic granulocyte percent (NEU%) 50-70 (%) | 50.1(39.25-58.95) | 59.95(54.33-63.63) | 0.068 |
| Neutrophilic granulocyte count (NEUT) 2-7 (10^9^/L) | 3.07(1.94-3.77) | 3.33(2.7-3.93) | 0.428 |
| Lymphocyte percent (LYMPH%) 20-40 (%) | 35.8(28.9-47.45) | 28.25(20.65-32.18) | 0.048 |
| Lymphocyte count (LYMN) 0.8-4.0 (10^9^/L) | 1.81(1.52-3.21) | 1.55(1.06-1.93) | 0.152 |
| Neutrophil/Lymphocyte Ratio | 1.41(0.82-2.06) | 2.09(1.74-3.09) | 0.069 |
| Monocytes count (MIDN) 0.12-1.0 (10^9^/L) | 0.53(0.35-0.71) | 0.6(0.4-0.64) | 0.871 |
| Erythrocyte count 0-1 (10^9^/L) | 4.9(4.62-5.23) | 4.68(4.3-4.89) | 0.14 |
| Hemoglobin (HB) 131-172 (g/L) | 150(128.5-154) | 130.5(119-152) | 0.332 |
| Platelet count (PLT) 85-303 (10^9^/L) | 229(161-253) | 155.5(69.25-246.5) | 0.425 |
| Prothrombin activity (PTA)75-100 (%) | 99(86.5-110.5) | 98.5(88-105.3) | 0.731 |
| Prothrombin time (PT)10-14(s) | 13.2(12.7-14.2) | 13.35(12.95-14) | 0.778 |
| Thrombocytocrit (PCT) 0.06-0.40 (%) | 0.23(0.17-0.25) | 0.19(0.15-0.32) | 0.93 |

^*^ Continuous variables were expressed in mean ± standard deviation (SD) or median (interquartile range [IQR]). Categorical variables were presented as number (percentage).

**Supplementary Table 3. All primers, antibodies and materials used in the manuscript.**

| REAGENT or RESOURCE | SOURCE | IDENTIFIER |
| --- | --- | --- |
| Antibodies | | |
| anti-β-actin | Abcam | ab8226 |
| anti-Caspase-3 | Abcam | ab32351 |
| anti-SLC39A1 | Abcam | ab105416 |
| anti-Cyclin-B1 | CST | #12231 |
| anti-Cyclin-D1 | CST | #55506 |
| anti-Cyclin-E1 | CST | #4129 |
| anti-CDK4 | Santa Cruz | sc-23896 |
| anti-CDK6 | Santa Cruz | sc-7961 |
| anti-mTOR | CST | #2983 |
| anti-p-mTOR | CST | #5536 |
| anti-ATG5 | CST | #2630 |
| anti-LC3 | MBL | PM036 |
| anti-Lamp1 | Santa Cruz | sc-20011 |
| anti-P62 | MBL | M162-3 |
| anti-MFN1 | CST | #14739 |
| anti-OPA1 | Abcam | ab157457 |
| anti-DRP1 | Abcam | ab184248 |
| anti-P-DRP1 | CST | #3455 |
| anti-FIS1 | Abcam | ab96764 |
| anti-MCU | CST | #14997 |
| anti-Cleaved Caspase-3 | CST | #9664 |
| anti-Cyto-C | CST | #4272 |
| anti-MFF | CST | #84580 |
| anti-HSP60 | CST | 4869S |
| anti-PINK1 | CST | #6946 |
| anti-Parkin | CST | #4211 |
| anti-Ki67 | Servicebio | GB111141 |
| anti-TGF-β | Servicebio | GB14154 |
| anti-GAPDH | Abcam | ab128915 |
| anti-BNIP3 | Abcam | Ab109362 |
| anti-FUNDC1 | Abcam | ab224722 |
| anti-TOMM20 | Servicebio | GB111481 |
| Bacterial and Virus Strains | | |
| DH5α | TIANGEN | CB101-02 |
| Chemicals, Peptides, and Recombinant Proteins | | |
| Zncl2 | Sigma | Z0152 |
| TPEN | MedChemExpress | HY-100202 |
| 3-methyladenine | APExBIO | A8353 |
| bafilomycin A1 | APExBIO | A8627 |
| MitoSox | MedChemExpress | HY-D1055 |
| TMRE | Beyotime | C2001S |
| Zinpry-1 | APExBIO | C4914 |
| Mdivi-1 | MedChemExpress | HY-15886 |
| pDsRed-endoplasmic reticulum | MiaoLingBio | P0141 |
| Peptide (SLC39A11-28):  MGPWGEPELLVWRPEAVASEPPVPVGLE | This paper | N/A |
| Oligonucleotides | | |
| shRNA targeting sequence: F: SLC39A1#1GCTGGCACCTTTCTCT  ATATCTTCAAGAGAGATATAGAGAAAGGTGCCAGC | This paper | N/A |
| shRNA targeting sequence F: SLC39A1#2 GGCCTGCTCTTCA  TCCAAATCTTCAAGAGAGATTTGGATGAAGAGCAGGCC | This paper | N/A |
| shRNA targeting sequence F: SLC66A1#1CACCGCCTGCAGAAA  TGGATCATCGTTCAAGAGACGATGATCCATTTCTGCAGGC | This paper | N/A |
| shRNA targeting sequence F: SLC66A1#2 GCCTACACCTGTG  CAACAAGATTCAAGAGATCTTGTTGCACAGGTGTAGGC | This paper | N/A |
| shRNA targeting sequence F: SLC50A1#1 GCTTTCTGGACTCG  CTCATTTTTCAAGAGAAAATGAGCGAGTCCAGAAAGC | This paper | N/A |
| shRNA targeting sequence F: SLC50A1#3 GCGCTTCAGACCC  TGTATATCTTCAAGAGAGATATACAGGGTCTGAAGCGC | This paper | N/A |
| Sequencing primer F:  SLC39A1 GCTGTTGCAGAGCCACCTTA | This paper | N/A |
| Sequencing primer F:  DRP1 GATGCCATAGTTGAAGTGGTGAC | This paper | N/A |
| Sequencing primer F: MCU TCCAGAAGCCAGAGACAGAC | This paper | N/A |
| Sequencing primer F:  SLC50A1 CATTTACGGAGCATGCGTGG | This paper | N/A |
| Sequencing primer F:  SLC66A3 CAGCTCCAGTGTCTGTGGAA | This paper | N/A |
| siRNA F: DRP1 CCCUAGCUGUAAUCACUAATT | This paper | N/A |
| siRNA F: MCU GUACGAAUUGAGAUUAGCATT | This paper | N/A |
| siRNA F: PINK1 CCTAACCGTCTCCGCTTCTTC | This paper | N/A |

**Supplementary Figure**

**

**

**Supplementary Figure 1. HCC samples from 12 non-relapsed and 15 relapsed HCC patients were collected for RNA sequencing.**

(A) A total of 78 significant differentially expressed genes (DEGs) were identified in 15 HCC relapsed group compared with 12 non-relapsed group after resection. Volcano plot for different expression genes in our HCC cohort. The red dots represent log10(P value) less than 0.05 and log2(FC values) greater than 1.5.

(B) The Kyoto Encyclopedia of Genes and Genomes (KEGG) and Gene Ontology (GO) analysis in 12 non-relapsed and the 15 relapsed HCC patients.

(C) Hallmark pathways was assessed from the RNA sequencing data in 12 non-relapsed and 15 relapsed HCC patients.

(D) Heatmap visualizes immune infiltration profiles in 12 non-relapsed and 15 relapsed HCC patients.

(E-F) Analysis of infiltrating immune cells, including B cells, NK cells, T cells in 12 non-relapsed and 15 relapsed HCC patients.

**
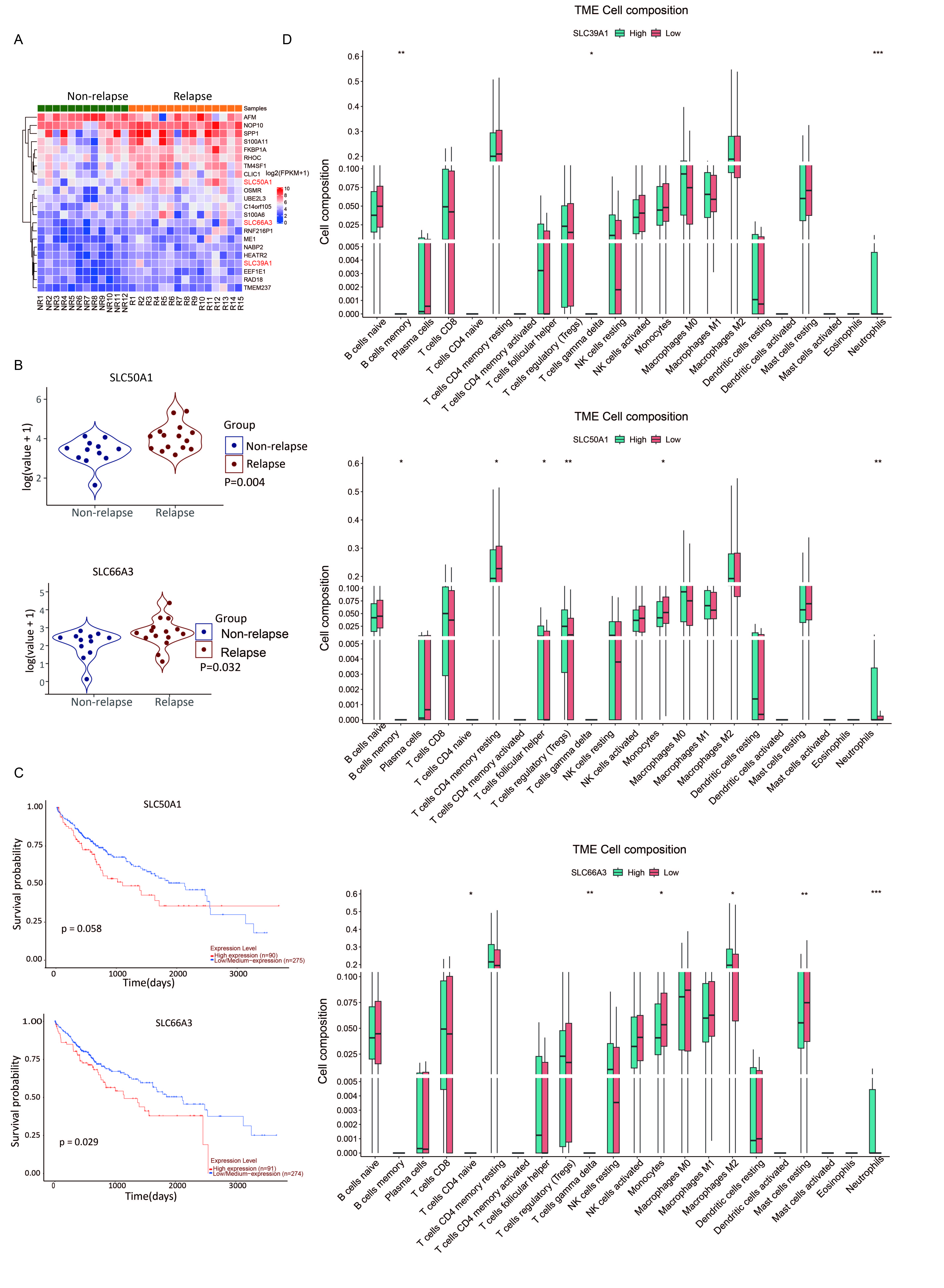
**

**Supplementary Figure 2. Immune infiltration profiles of SLC39A1, SLC50A1, and SLC66A3.**

(A) Heatmap visualizes the DEGs between12 non-relapsed and 15 relapsed HCC patients.

(B) SLC50A1 and SLC66A3 mRNA expressions in 12 non-relapsed and 15 relapsed HCC patients in our HCC cohort.

(C) Kaplan-Meier curves of overall survival of HCC patients according to the level of SLC50A1 and SLC66A3 in TCGA dataset.

(D) TME cell composition of SLC39A1, SLC50A1 and SLC66A3 in TCGA-LIHC dataset.

***P< 0.001, **P< 0.01, and *P< 0.05.


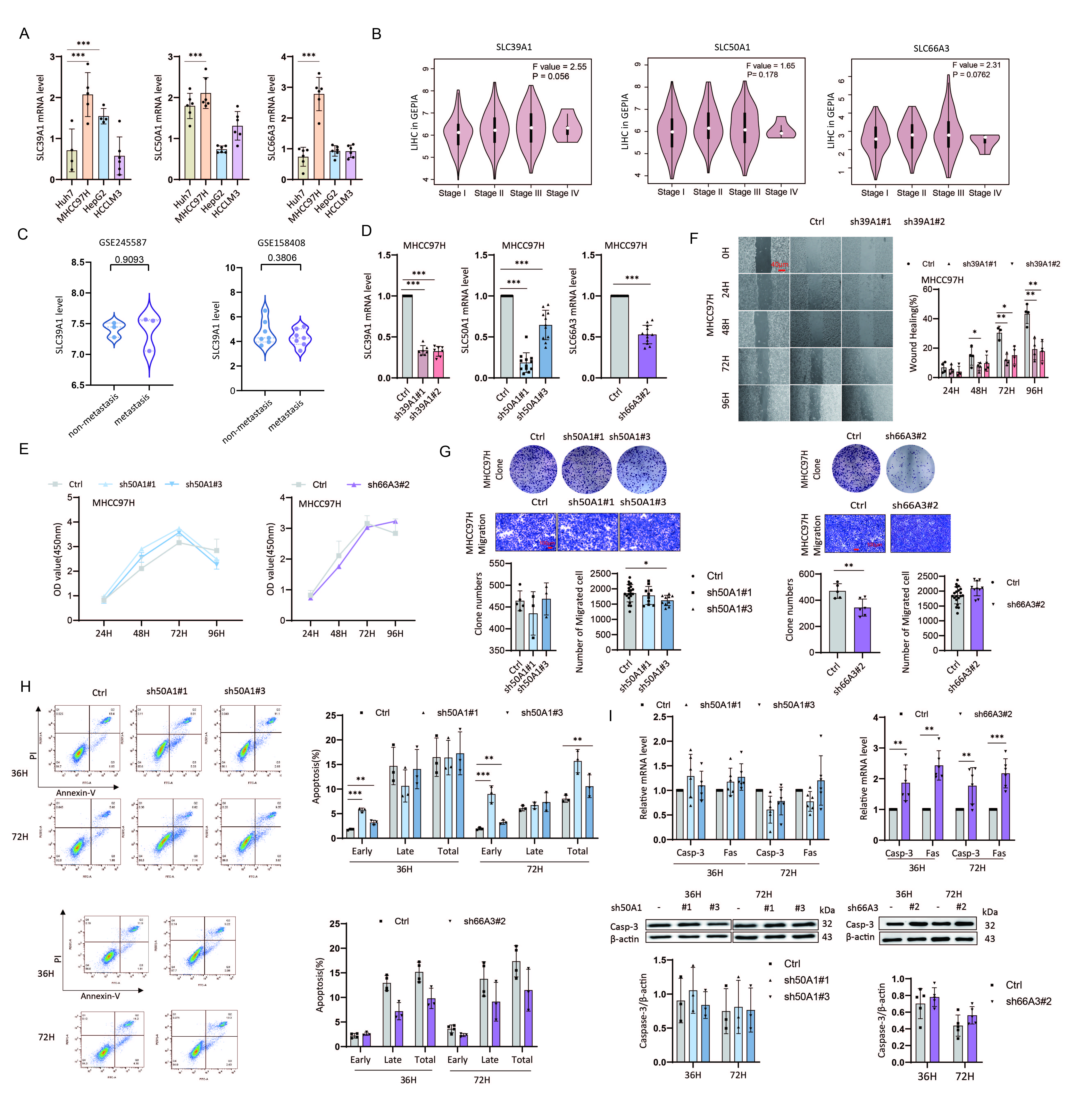


**Supplementary Figure 3. Analysis of cell proliferation, migration, invasion and cell apoptosis in SLC50A1 and SLC66A3 knockdown cells.**

(A) The mRNA level of SLC39A1, SLC50A1 and SLC66A3 were detected in Huh7, MHCC97H, HepG2 and HCCLM3 cell lines.

(B) The expression of SLC39A1, SLC50A1 and SLC66A3 in different tumor stages in LIHC GEPIA dataset.

(C) The expression of SLC39A1 between metastatic and non-metastatic HCC patient in LIHC GEPIA dataset.

(D) The expression of SLC39A1, SLC50A1 and SLC66A3 were detected in MHCC97H cells.

(E) Cell proliferation analysis by CCK8 assays in MHCC97H cells.

(F) Cell migration analysis by wound healing assay in MHCC97H cells.

(G) Cell clonal formation and cell migration in MHCC97H cells by plate clone formation and transwell assays.

(H) Cell apoptosis analysis by flow cytometry with PI/Annexin V staining in MHCC97H cells.

(I) The mRNA expressions of Casp-3 and Fas and the protein level of Casp-3 in MHCC97H cells.

Data are expressed as mean ± SD(n≥3). ***P< 0.001, **P< 0.01, and *P< 0.05.


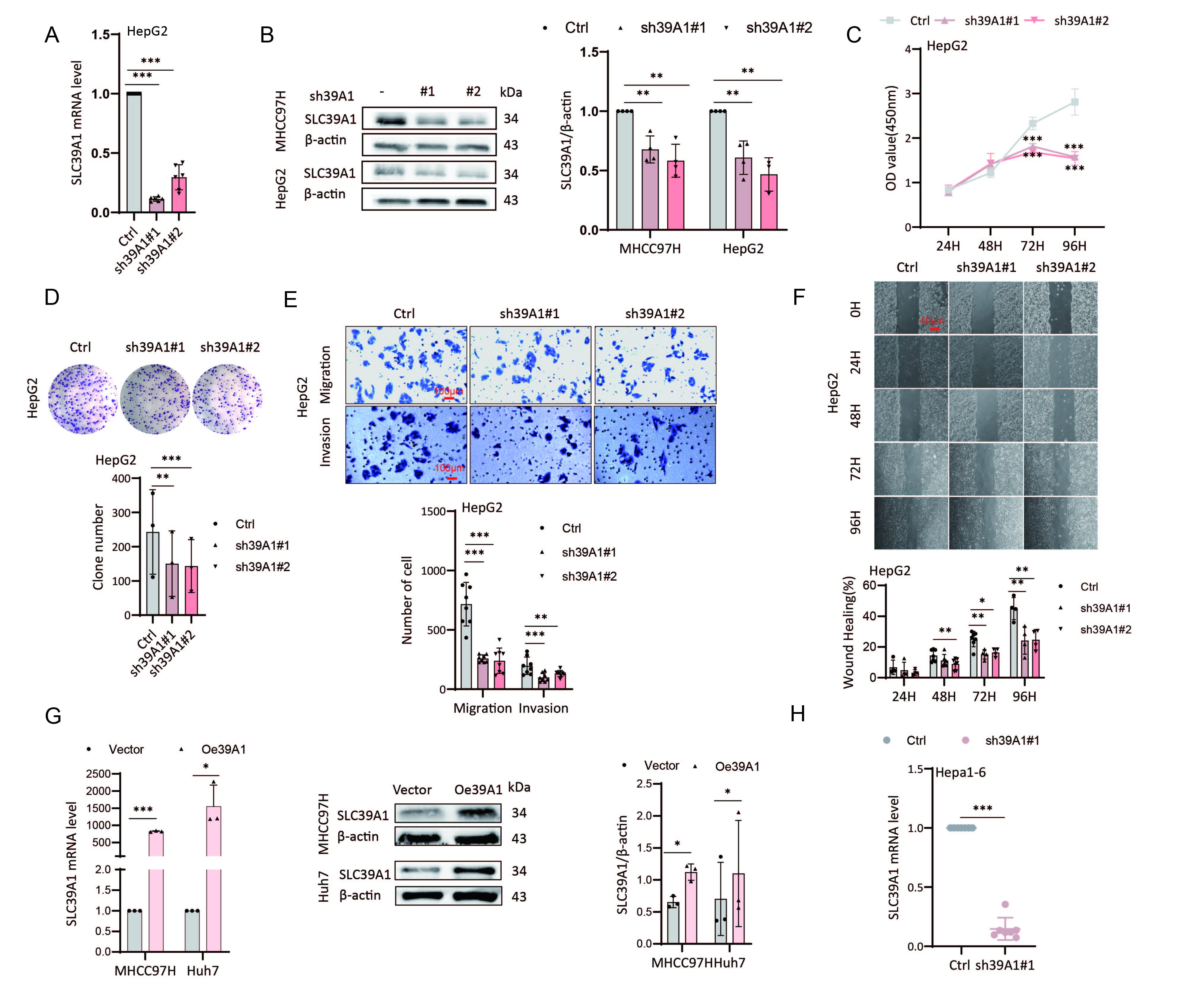


**Supplementary Figure 4.** **SLC39A1 promotes cell proliferation, migration and invasion in HepG2 cells.**

(A) The expression of SLC39A1 in HepG2 cells.

(B) The protein of SLC39A1 in SLC39A1 knockdown HepG2 and MHCC97H cells.

(C) Cell proliferation analysis by CCK8 assays in SLC39A1 silenced cells.

(D) Cell proliferation analysis by plate clone formation assays in SLC39A1 knockdown cells.

(E) Cell migration and invasion analysis by transwell assays in SLC39A1 silenced cells.

(F) Cell migration analysis by wound healing assay in SLC39A1 silenced cells.

(G) The level of SLC39A1 in SLC39A1 overexpressed cells.

(H) The expression level of SLC39A1 in Hepa1-6 cells.

Data are expressed as mean ± SD(n≥3). ***P< 0.001, **P< 0.01, and *P< 0.05.


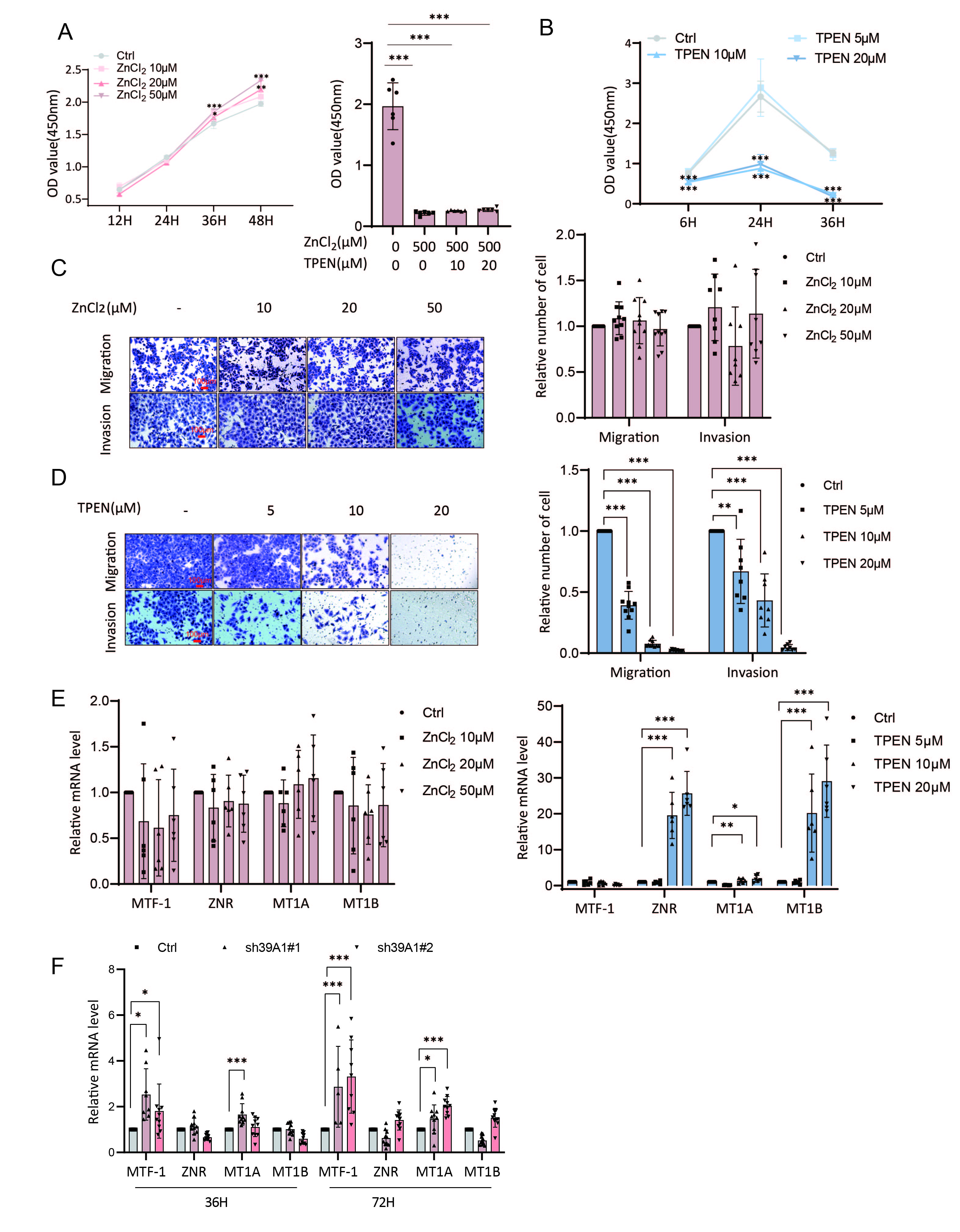


**Supplementary Figure 5. Impact of intracellular Zn^2+^ on cell proliferation and migration.**

(A-B) The cell proliferation analysis in MHCC97H cells treated with different combinations of ZnCl_2_ and TPEN.

(C-D) The cell migration and invasion analysis by transwell assays in MHCC97H cells treated with different combinations of ZnCl_2_ and TPEN.

(E) Expressions of MTF-1A, ZNR, MT1A/B in MHCC97H cells treated with different combinations of ZnCl_2_ and TPEN.

(F) The mRNA expression of MTF-1A, ZNR, MT1A/B in control and SLC39A1-knockdown cells.

Data are expressed as mean ± SD(n≥3). ***P< 0.001, **P< 0.01, and *P< 0.05.

**
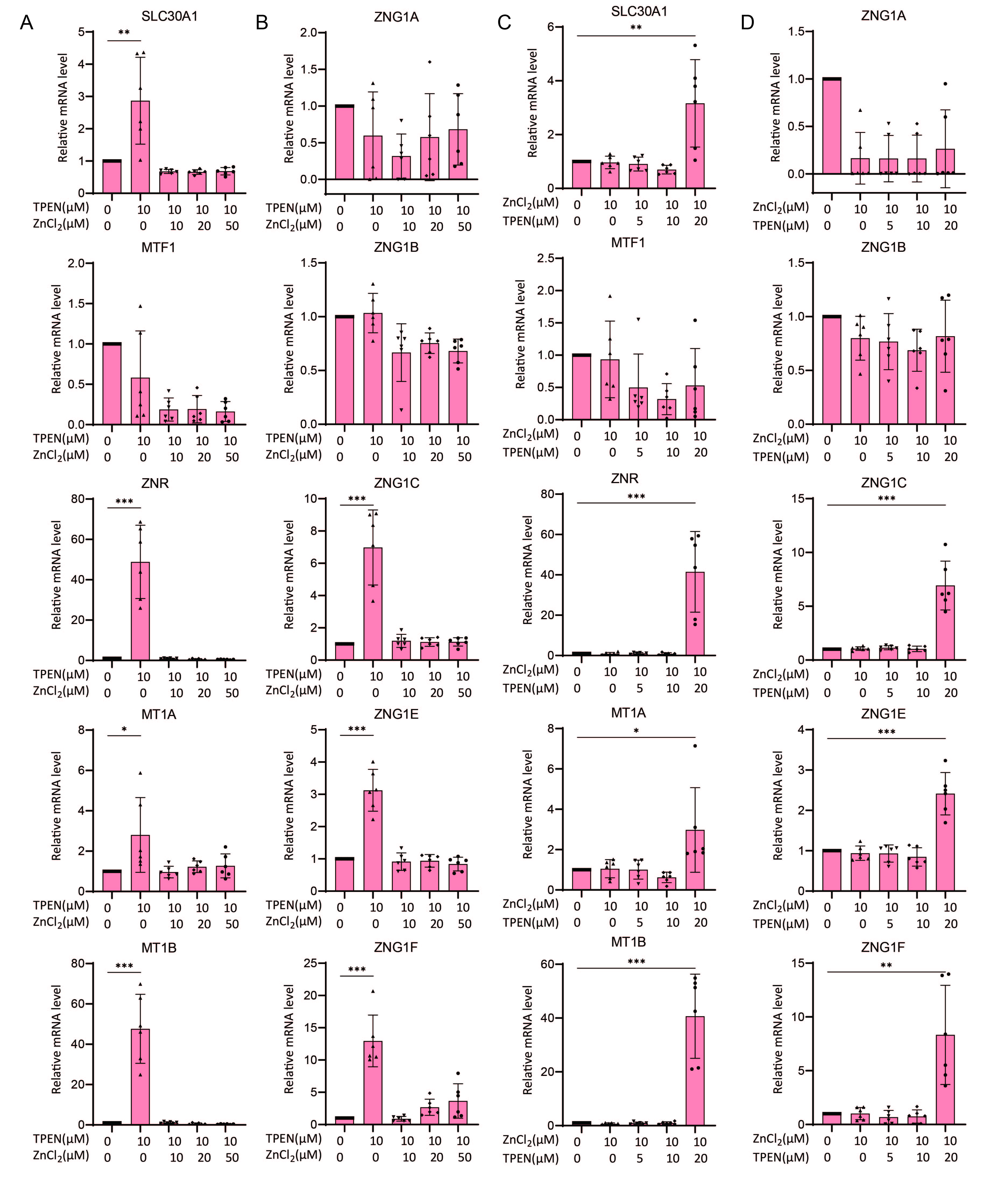
**

**Supplementary Figure 6. The transcripts levels of key elements in maintaining Zn^2+^ homeostasis were** **demonstrated** **in MHCC97H cells in various combination of ZnCl2 and TPEN.**

Data are expressed as mean ± SD(n≥3). ***P< 0.001, **P< 0.01, and *P< 0.05.


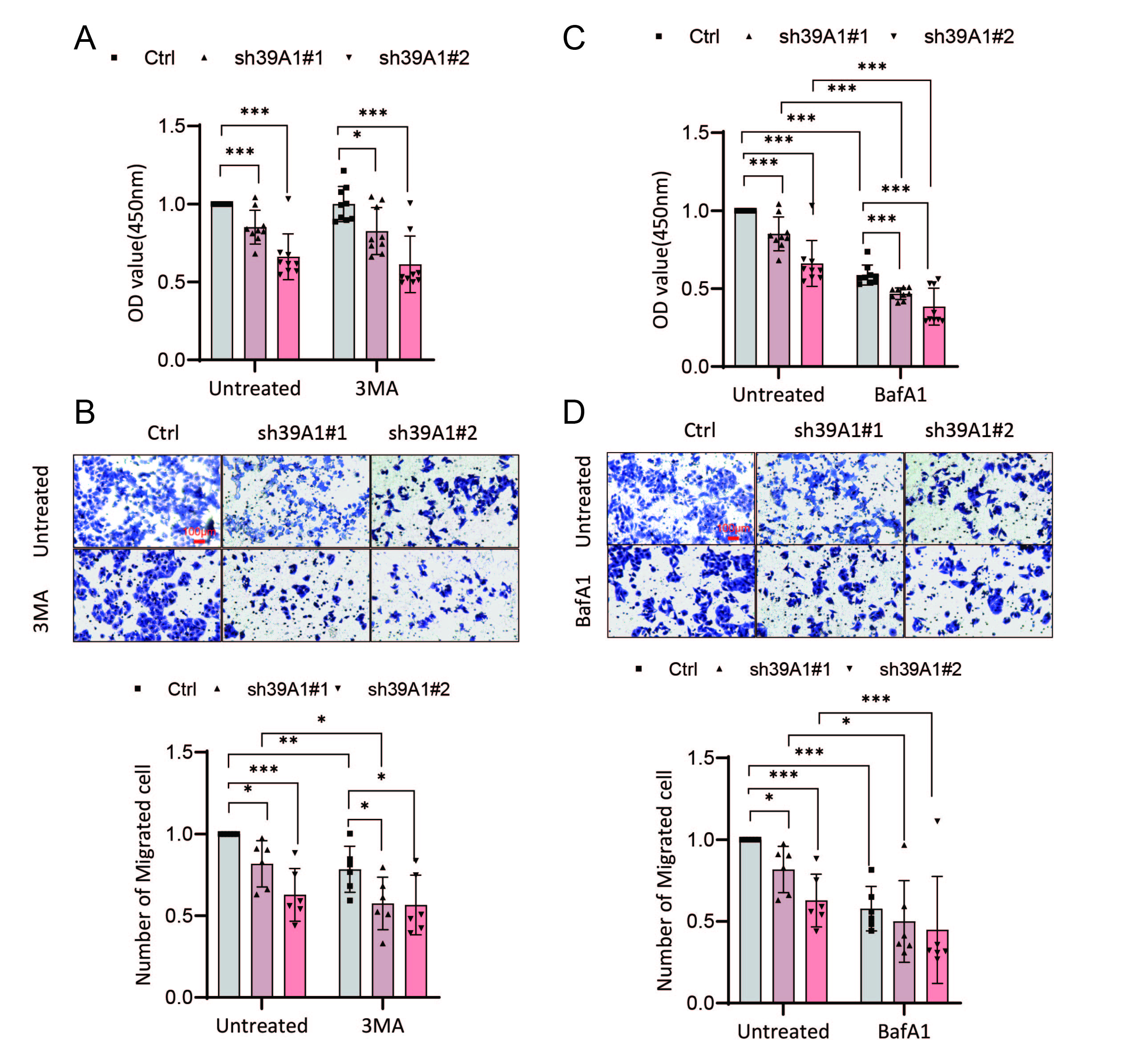


**Supplementary Figure 7. Treatment with 3-MA and BafA1 failed to reversed inhibitory effect of cell proliferation.**

(A-D) Cell proliferation and cell migration analysis by CCK8 and transwell assays in SLC39A1 silenced cells with or without 3MA/BafA1 treatment.

Data are expressed as mean ± SD(n≥3). ***P< 0.001, **P< 0.01, and *P< 0.05.


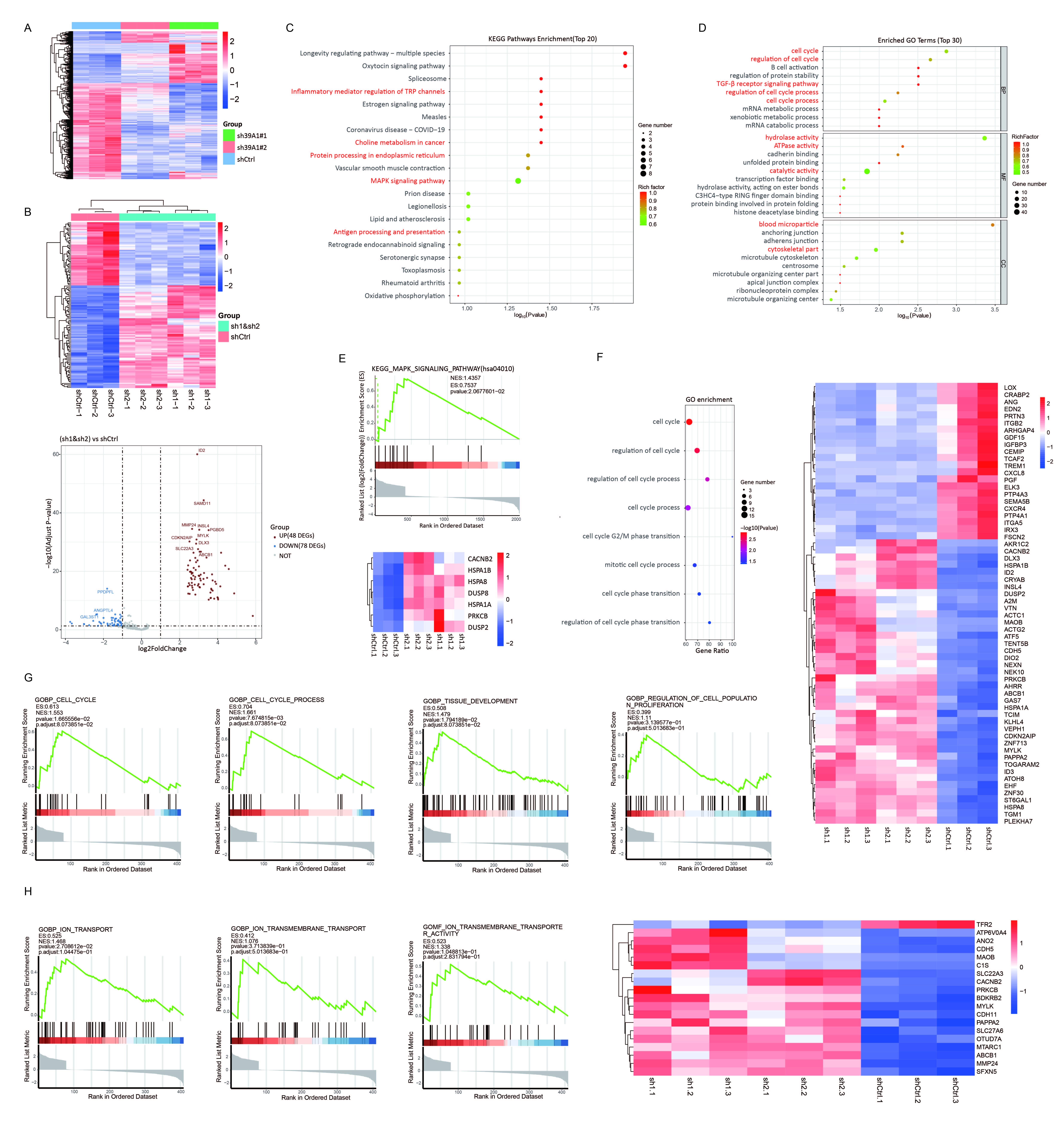


**Supplementary Figure 8. RNA** **sequencing analysis of shSLC39A1 in MHCC97H cells** **compared to control cells.**

(A-B) Heatmap and Volcano plot visualize the differential gene expression profiles. The DEGs were obtained from the intersection of correlated genes in the shSLC39A1#1 and shSLC39A1#2 groups versus control group.

(C) TOP 20 pathways in SLC39A1 silenced cells were enriched by KEGG analysis.

(D) GO analysis in SLC39A1 silenced cells compared to control cells.

(E) MAPK signaling pathway was enriched by KEGG analysis.

(F-H) Cell cycle related biological processes (F-G) and ion transport pathways (H) in SLC39A1 silenced cells were enriched by GO analysis.

**
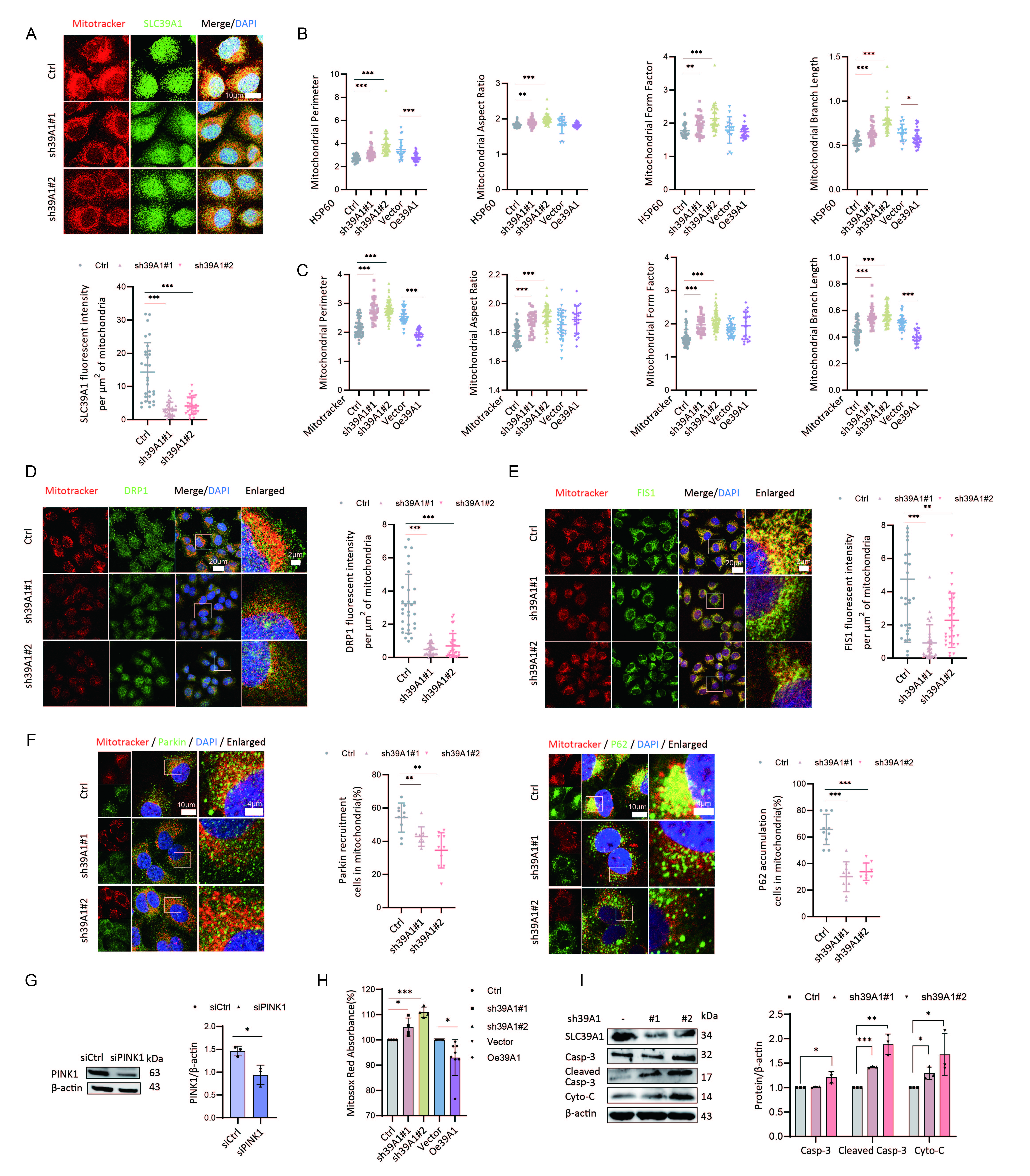
**

**Supplementary Figure 9. SLC39A1 was located in mitochondrion and promotes mitochondrial fission accompanied by inhibition cell apoptosis.**

(A) Immunofluorescent staining for SLC39A1 and Mitotracker staining in MHCC97H cells (n=30 cells).

(B-C) Mitochondrial morphology was assessed by HSP60 antibody and Mitotracker staining, including mitochondrial perimeter, aspect ratio, form factor and branch length (n=30-80 cells).

(D-E) Representative images of DRP1, FIS1 (Green) and Mito-tracker Red by immunoﬂuorescence in MHCC97H cells (n=30 cells).

(F) Immunofluorescent staining for Parkin, P62 and Mitotracker staining in MHCC97H cells (n=90-150 cells).

(G) The protein of PINK1 in MHCC97H cells.

(H) Intracellular ROS detected by MitoSox Red staining in MHCC97H cells.

(I) Western blot analysis of Casp-3, Cleaved Casp-3 and Cytochrome C in MHCC97H cells.

Data are expressed as mean ± SD. ***P< 0.001, **P< 0.01, and *P< 0.05.

**
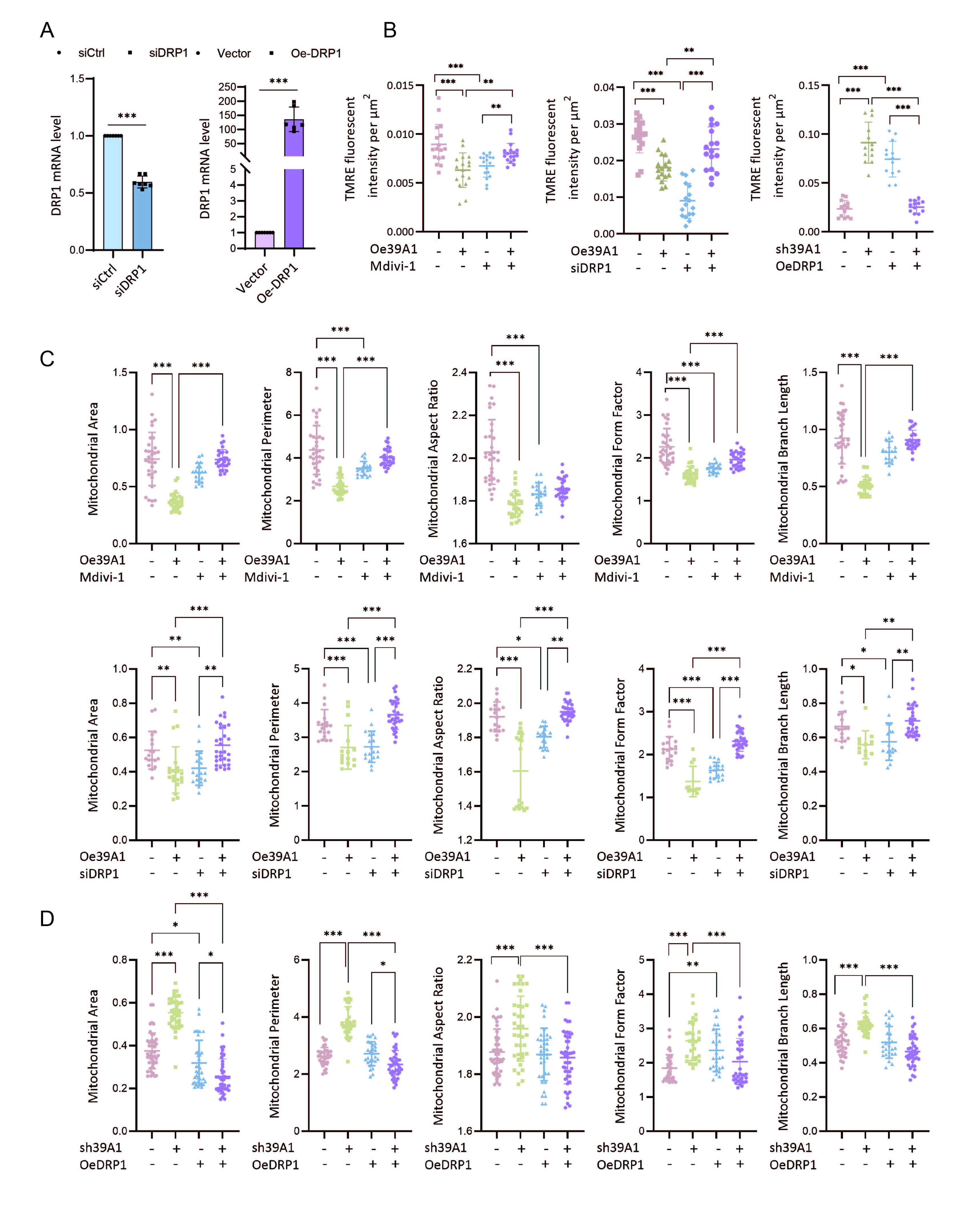
**

**Supplementary Figure 10. Interaction between DRP1 and SLC39A1 contributes to MMP reduction and mitochondrial fragmentation**

(A) The transcripts level of DRP1 were detected by RT-qPCR.

(B) Quantitative analysis of TMRE fluorescence in SLC39A1 overexpressed cells and SLC39A1 silenced cells. (n=30-80 cells)

(C-D) Quantitative analysis of mitochondrial morphology in SLC39A1 overexpressed cells and SLC39A1 silenced cells, including mitochondrial area, perimeter, aspect ratio, form factor and branch length (n=30-80 cells).

Data are expressed as mean ± SD. ***P< 0.001, **P< 0.01, and *P< 0.05.

**
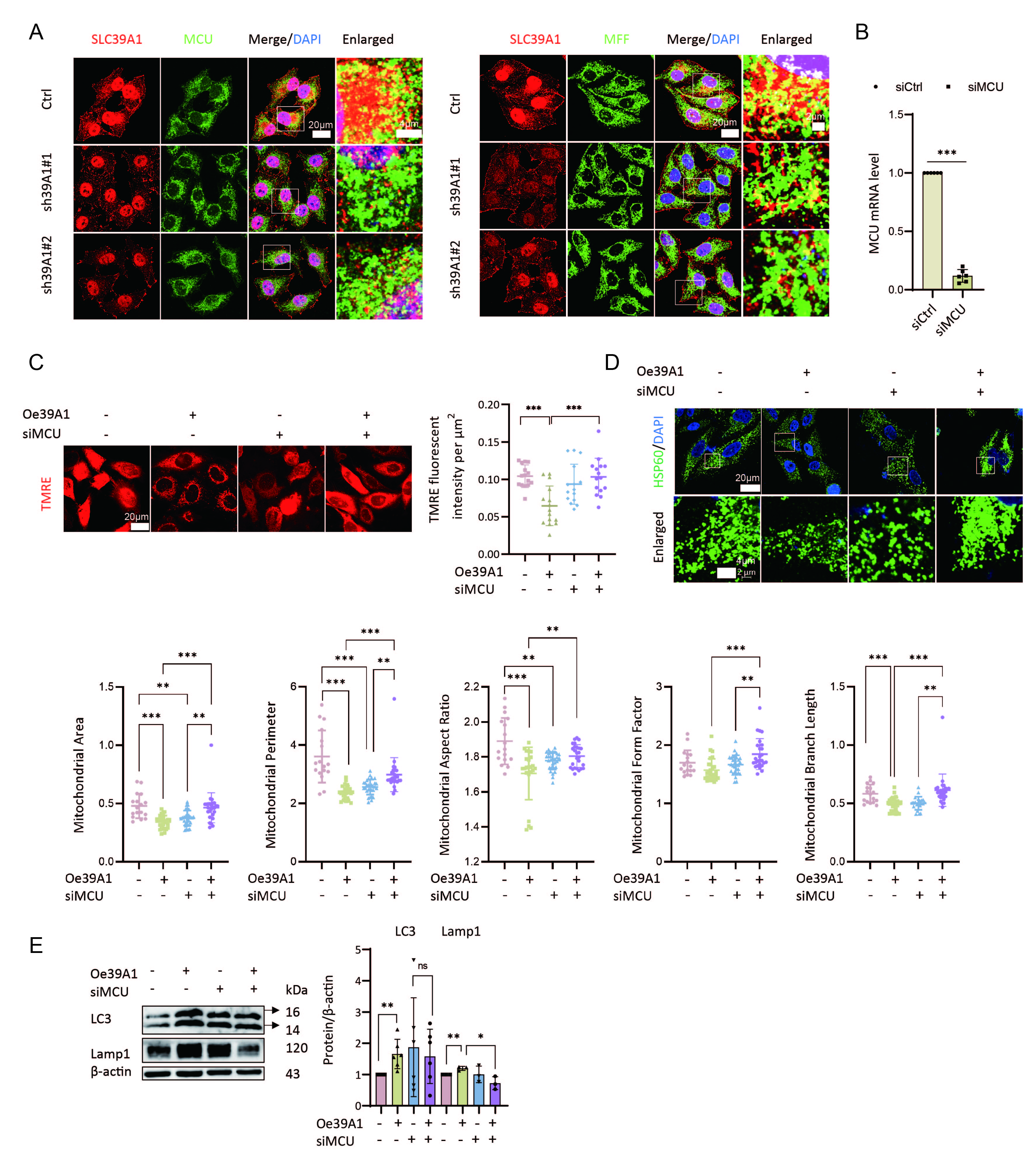
**

**Supplementary Figure 11. SLC39A1-MCU axis contributes to MMP reduction and mitochondrial fragmentation**

(A) Immunoﬂuorescence staining of MCU, MFF (Green) and SLC39A1 (Red) in MHCC97H cells.

(B) The transcripts level of MCU was detected by RT-qPCR.

(C-D) Quantitative analysis of TMRE fluorescence and mitochondrial morphology in SLC39A1 overexpressed cells. (n=30-80 cells)

(E) Western blot analysis of LC3 and LAMP1 in MHCC97H cells treated with genetic inhibition of MCU (siMCU).

Data are expressed as mean ± SD(n≥3). ***P< 0.001, **P< 0.01, and *P< 0.05.


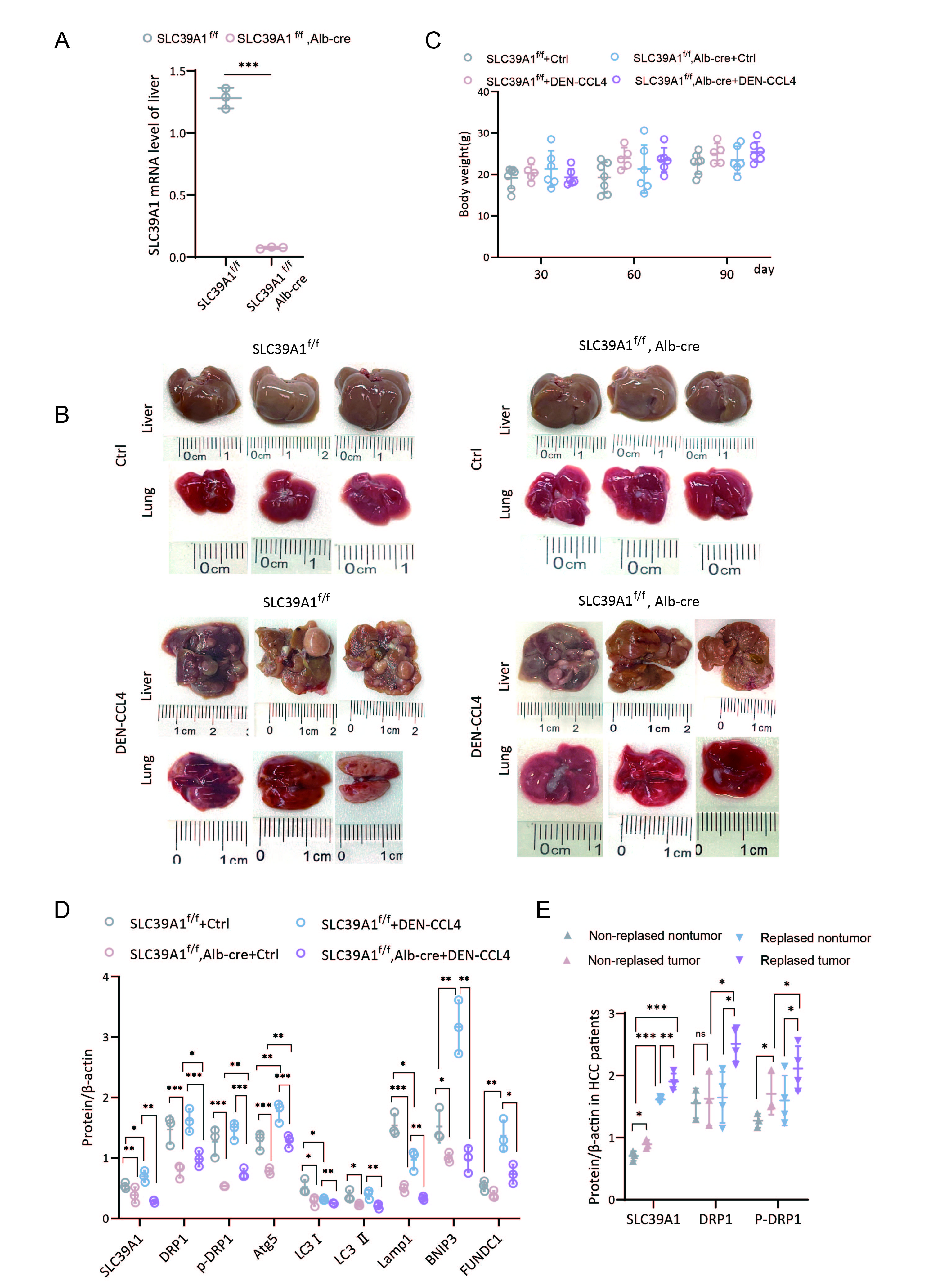


**Supplementary Figure 12. Genetic knockout of SLC39A1 attenuates DEN-CCL4-driven HCC development and represses autophagy in mice.**

(A) The transcripts level of SLC39A1 of liver tissues was detected in SLC39A1^f/f^,Alb-Cre and SLC39A1^f/f^ mice. (Ctrl: SLC39A1^f/f^ n=3, SLC39A1^f/f^,Alb-Cre n=3)

(B) Representative macroscopic images of liver and lung from DEN-CCL4 models and control models. (Ctrl: SLC39A1^f/f^ n=3, SLC39A1^f/f^,Alb-Cre n=3; DEN-CCL4: SLC39A1^f/f^ n=3, SLC39A1^f/f^,Alb-Cre n=3).

(C) Body weight of SLC39A1^f/f^,Alb-Cre and SLC39A1^f/f^ with DEN-CCL4 intraperitoneal injection in C57BL/6J mice. (Ctrl: SLC39A1^f/f^ n=7, SLC39A1^f/f^,Alb-Cre n=6; DEN-CCL4: SLC39A1^f/f^ n=5, SLC39A1^f/f^,Alb-Cre n=6).

(D) Statistical analysis of DRP1, Atg5, LC3, Lamp1, BNIP3 and FUNDC1of liver tissues in SLC39A1^f/f^,Alb-Cre and SLC39A1^f/f^ mice (n=3).

(E) Statistical analysis of SLC39A1, DRP1 and P-DRP1in HCC non-relapsed (n=3) and relapsed (n=4) patients. NT: adjacent tissues; T: tumor tissues.

Data are expressed as mean ± SD(n≥3). ***P< 0.001, **P< 0.01, and *P< 0.05.

**
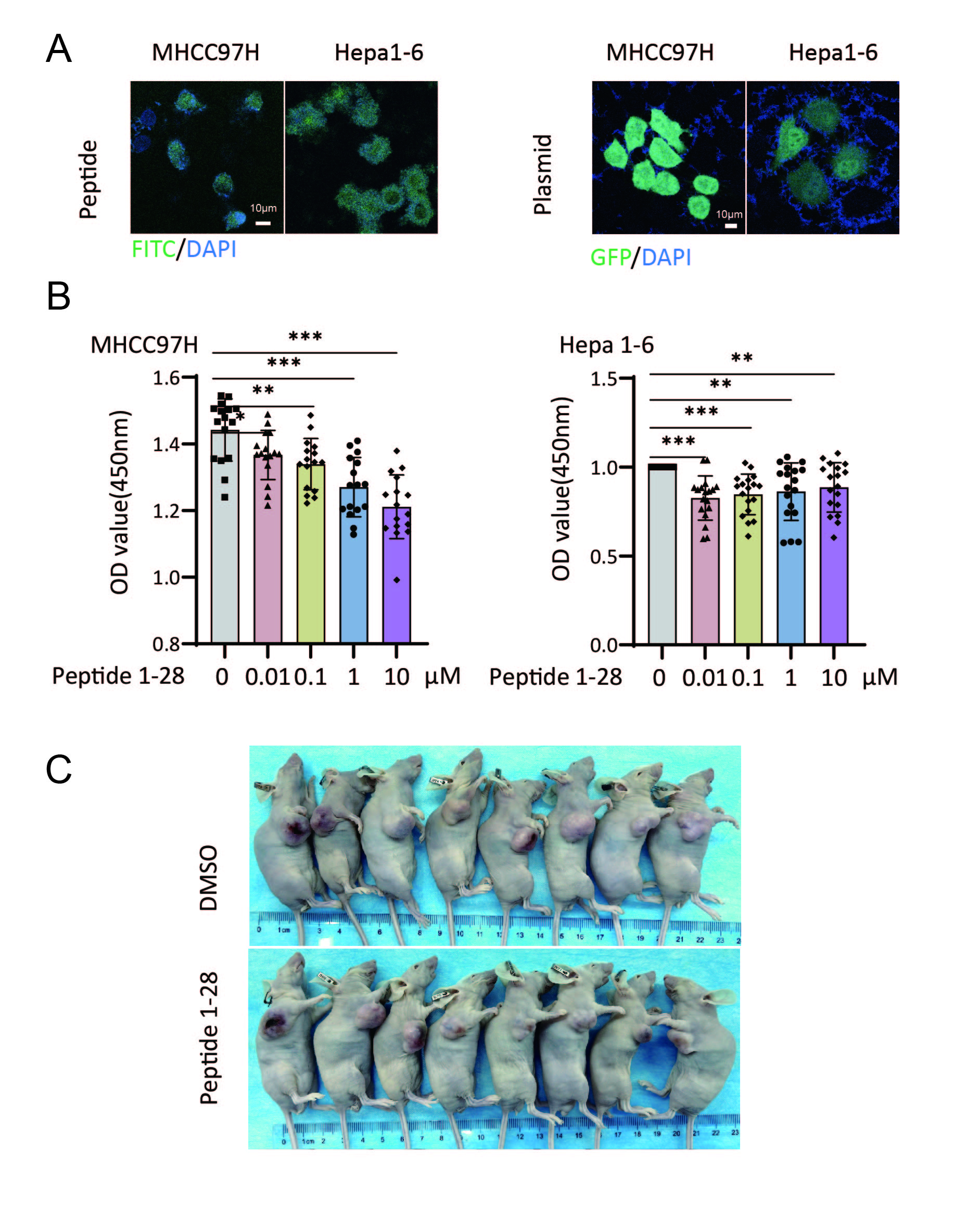
 Supplementary Figure 13. Specific plasmid and peptide targeting SLC39A1 inhibit cell proliferation and tumor growth.**

(A) Immunoﬂuorescent staining of specific peptide and plasmid targeting SLC39A1 in MHCC97H and Hepa1-6 cells.

(B) Cell proliferation analysis in both MHCC97H and Hepa1-6 cells treated with specific peptide targeting SLC39A1.

(C) Tumor volumes and cell proliferation in mice by subcutaneous injection with Hepa1-6 cells followed by treatment with DMSO or specific peptide targeting SLC39A1 (2.5mg/g) through intratumoral injection daily for 10 days (n=8).

Data are expressed as mean ± SD(n≥3). ***P< 0.001, **P< 0.01, and *P< 0.05.
